# Benzodiazole-Based Covalent Organic Frameworks for Enhanced Photocatalytic Dehalogenation of Phenacyl Bromide Derivatives

**DOI:** 10.3390/polym16182578

**Published:** 2024-09-12

**Authors:** Ming Wang, Jiaying Qian, Shenglin Wang, Zhongliang Wen, Songtao Xiao, Hui Hu, Yanan Gao

**Affiliations:** 1 Key Laboratory of Ministry of Education for Advanced Materials in Tropical Island Resources, Hainan University, No. 58, Renmin Avenue, Haikou 570228, Chinaygao@hainanu.edu.cn (Y.G.); 2 China Institute of Atomic Energy, Beijing 102413, China

**Keywords:** covalent organic frameworks, heteropore, heterogeneous photocatalysis, dehalogenation

## Abstract

Covalent organic frameworks (COFs) have garnered significant interest within the scientific community due to their distinctive ability to act as organic semiconductors responsive to visible light. This unique attribute makes them up-and-coming candidates for facilitating photocatalytic organic reactions. Herein, two donor–acceptor COFs, TPE-BSD-COF and TPE-BD-COF, have been designed and synthesized by incorporating electron-rich tetraphenylethylene and electron-deficient benzoselenadiazole and benzothiadiazole units into the framework through a Schiff-base polycondensation reaction. Both COFs exhibit exceptional crystallinity and enduring porosity. TPE-BSD-COF and TPE-BD-COF exhibit broad light absorption capabilities, a narrow optical band gap, and low electrochemical impedance spectrum (EIS) levels, indicating that the two COFs are effective heterogeneous photocatalysts for the reductive dehalogenation of phenacyl bromide derivatives under blue LED irradiation. A high photocatalytic yield of 98% and 95% was achieved by TPE-BSD-COF and TPE-BD-COF catalysts, respectively, within only one hour.

## 1. Introduction

Over the past few years, the adoption of heterogeneous photocatalysis in the field of organic synthesis has gained substantial momentum. This technique is celebrated for its straightforward application, eco-friendly processes, and mild operational conditions. As such, it is widely regarded as an effective and sustainable strategy for generating high-value fine chemicals [1,2,3,4]. In this area of study, creating and developing photocatalysts with outstanding performance in photocatalysis continues to be a significant obstacle. Significant efforts have been dedicated to developing highly active photocatalysts, including inorganic semiconductor materials [5,6], organometallic compounds [7], and organic dye molecules [8]. All these materials exhibit strong light absorption and excellent redox characteristics when excited by light at appropriate wavelengths. In recent years, the development of novel photocatalysts has focused on polymeric systems, such as polyaniline (PANI) [9], polythiophene [10,11], graphitic carbon nitride (g-C_3_N_4_) [12,13], polypyrrole (PPy) [14], and metal–organic frameworks (MOFs) [15,16,17]. These heterogeneous photocatalysts have exhibited high catalytic activities in various organic transformations and have such advantages over homogeneous counterparts as low cost, easy separation, and high reusability. Covalently linked porous aromatic frameworks (PAFs) have emerged as a promising platform as metal-free and highly efficient photocatalysts and have demonstrated tremendous potential in various organic syntheses under light irradiation conditions [18,19].

Covalent organic frameworks (COFs), as a new class of crystal porous organic polymers, have a predictable topology and permanent pores. The unique advantage of COFs lies in their ease of structural and functional tuning [20,21,22,23]. Thus, COFs are excellent materials for applications in adsorption [24,25], sensing [26,27], energy storage [28], and heterogeneous catalysis [29,30,31]. Two-dimensional (2D) COFs showcase regular columnar π-arrays and one-dimensional (1D) nanopores arising from the stacked layers of 2D macromolecular sheets. These organized and conductive columns provide a prime route for photogenerated electron–hole pairs’ separation, diffusion, and movement. Therefore, from a structural perspective, photoactive 2D COFs can be strong candidates for optoelectronic materials and photocatalysts [32,33]. The pioneering work by Lotsch’s group has reported a hydrazone-based 2D COF that catalyzed hydrogen production from water under visible light [34]. Nonetheless, the use of 2D COFs as photocatalysts for the reductive dehalogenation reaction of phenacyl bromide has been limited [35,36].

Benzoselenadiazole and benzothiadiazole compounds are renowned for their photosensitizing properties and are commonly utilized in organic light-emitting diodes (OLEDs) and photovoltaic cells [37]. Recently, the development of COFs incorporating benzoselenadiazole/benzothiadiazole units has surged, revealing exceptional photocatalytic capabilities [38,39]. Furthermore, heteropore COFs offer various advantages compared to COFs with uniform single porosity; however, there is a lack of reports on heteropore COFs in the photocatalysis field [40]. In this work, we synthesized two heteropore COFs, namely TPE-BSD-COF and TPE-BD-COF, that were constructed by a photoactive benzoselenadiazole and benzothiadiazole as a building block. The two COFs demonstrate outstanding photocatalytic performance due to their high crystallinity, intrinsic porosity, and low EIS. Acting as a heterogeneous photocatalyst for the reductive dehalogenation of phenacyl bromide derivatives, a photocatalytic yield of 98% and 95% catalyzed by the novel TPE-BSD-COF and TPE-BD-COF, respectively, was achieved within just one hour.

## 2. Materials and Methods

Materials: All chemical reagents, unless otherwise noted, were purchased directly from Aladdin and TCI without the need for further purification.

Synthesis of TPE-BSD-COF: 4,4′,4″,4‴-(Ethene-1,1,2,2-tetrayl)tetraaniline (TPE, 9.8 mg, 0.025 mmol) and 4,4′-(benzoselenadiazole-4,7-diyl)dibenzaldehyde (BSD, 20.0 mg, 0.05 mmol) were taken in a sealed tube and a mixture of *o*-DCB/*n*-butanol (2 mL, *v*/*v* 1/1) and 3M acetic acid (0.1 mL) was added into the tube. The tube was sonicated for five minutes and degassed via a freeze–pump–thaw process. Then, the tube was sealed, allowing the reaction to proceed at 120 °C for 3 days. The precipitate was collected, washed with THF, and dried under vacuum to yield a yellow COF powder in 79% isolated yield.

Synthesis of TPE-BD-COF TPE: TPE-BD-COF TPE was synthesized according to a reported procedure [41]. Typically, TPE (9.8 mg, 0.025 mmol) and 4,4′-(benzothiadiazole-4,7-diyl)dibenzaldehyde (BD, 17.8 mg, 0.05 mmol) were taken in a sealed tube. A mixture of *o*-DCB/*n*-butanol (2 mL, *v*/*v* 1/1) and 3M acetic acid (0.1 mL) was added into the tube. The mixture was sonicated for 5 min and then degassed via a freeze–pump–thaw process. The tube was sealed and allowed the reaction to proceed at 120 °C for 3 days. The precipitate was collected, washed with THF, and dried under vacuum to yield a yellow COF powder in 82% isolated yield.

Characterizations: All the reagents and solvents were of reagent grade and used as received. ^1^H NMR spectra and ^13^C NMR spectra were recorded on a Bruker DPX 400 spectrometer (Bruker, Zurich, Switzerland). Solid-state ^13^C CP/MAS NMR was performed on a JNM-ECZ600R 600 MHz spectrometer (JEOL, Tokyo, Japan) with a 3.2 mm double-resonance MAS probe. The FT-IR spectra were obtained using a JASCO FT-IR-6800 Fourier transform infrared spectrophotometer (JASCO, Tokyo, Japan). Solid UV–vis spectra were recorded on a JASCO V-770 Spectrophotometer (JASCO, Tokyo, Japan) within the wavelength range of 200–800 nm. Thermogravimetric analysis (TGA) was recorded on a TG-DTA8122 thermal analyzer with an N_2_ flow rate of 30 mL min^−1^ at a heating rate of 5 °C min^−1^ from room temperature to 800 °C. Powder X-ray diffraction (PXRD) data were collected on a Rigaku Smart Lab powder diffractometer using a Cu Kα source (λ = 1.5418 Å) over the range of 2θ = 2.0–30.0° with a step size of 0.02° and 2 s per step. The sorption isotherms for N_2_ were measured using a MICROTRAC MRB BELSORP MAX II analyzer (MicrotracBEL, Tokyo, Japan) with ultra-high-purity gas (99.999% purity). To estimate the pore size distributions, nonlocal density functional theory (NLDFT) was applied to analyze the N_2_ isotherm on the basis of the model of N_2_@77K on carbon with slit pores and the method of non-negative regularization. The scanning electron microscopy (SEM) images were obtained on a thermoscientific Verios G4 UC scanning electron microscope (Thermo Scientific, Waltham, MA, USA). Low-dose high-resolution transmission electron microscopy (HRTEM) was performed on a thermoscientific Talos F200X G2 (Thermo Scientific, Waltham, USA). The photoluminescence spectroscopy (PL) was collected on a JASCO FP-8600 (JASCO, Tokyo, Japan). The fluorescence lifetime was collected on a Quantaurus-Tau C16361 (Hamamatsu Photonics Co., Ltd., Hamamatsu, Japan).

## 3. Results and Discussion

### 3.1. Synthesis and Characterization of COFs

**Figure 1:**
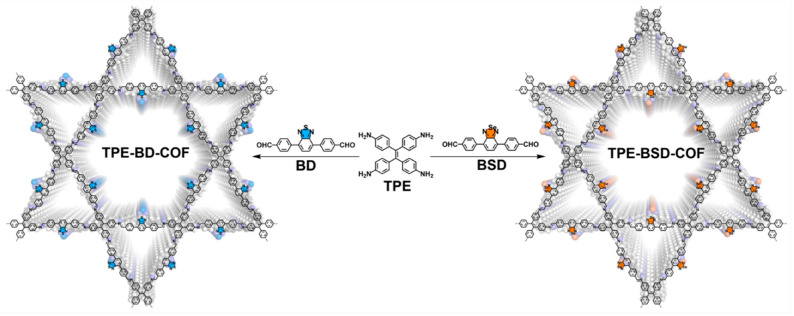
Schematic diagram of synthesized TPE-BSD-COF and TPE-BD-COF.

A novel benzoselenadiazole-based imine-linked framework, designated as TPE-BSD-COF, was successfully synthesized through the condensation reaction between TPE and BSD monomers (Figure 1 and Figure S1). The solvothermal synthesis was carried out in a mixed solvent of o-dichlorobenzene and 1-butanol, with acetic acid (6 M) as catalyst at 120 °C over 72 h. Upon completion, a yellow powder was produced with a yield of 79%. The detailed synthesis procedure was provided in the Supporting Information. The synthesis of TPE-BD-COF was carried out according to reported literature (Figure 1 and Figure S2) [41].

**Figure 2:**
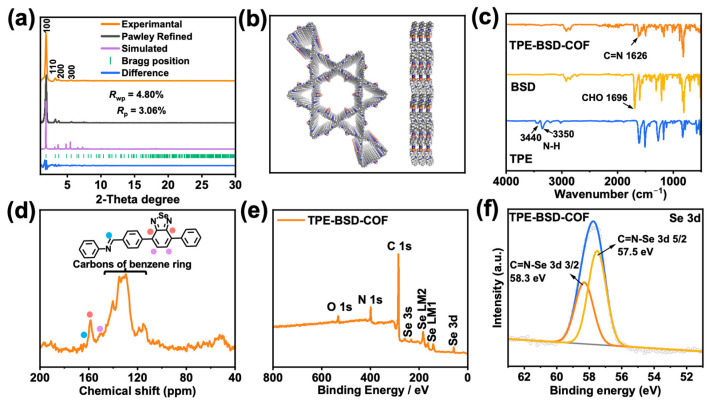
The experimental and simulated PXRD patterns of TPE-BSD-COF (**a**). Structural model of TPE-BSD-COF (**b**). FT-IR spectra of TPE-BSD-COF and the monomers (**c**). Solid-state ^13^C CP/MAS NMR spectra of TPE-BSD-COF (**d**). Survey-scan XPS spectrum (**e**) and high-resolution Se 3d XPS spectrum (**f**) of TPE-BSD-COF.

Powder X-ray diffraction (PXRD) analysis revealed that both TPE-BSD-COF and TPE-BD-COF are crystalline porous materials (Figure 2a and Figure S3a). The PXRD pattern of TPE-BSD-COF exhibits a significant peak at 1.82° as well as smaller peaks at 3.15°, 3.64°, and 4.82°, corresponding to the (100), (110), (200), and (300) reflections, respectively. The experimental PXRD pattern of TPE-BSD-COF corresponded well with the pattern calculated in AA stacking mode (Figure 2a, purple line, Figure 2b), while showing poor agreement with the pattern calculated in AB stacking mode (Figure S4). This indicates that TPE-BSD-COF possesses a hexagonal topology characterized by AA stacking. The Pawley refined lattice parameters of TPE-BSD-COF were *a* = *b* = 55.98 Å, *c* = 5.04 Å, and *α* = *β* = *γ* = 90°; the corresponding *R*_wp_ and *R*_p_ values were 4.80% and 3.06%, respectively. TPE-BD-COF exhibits distinct peaks at 1.82°, 3.64°, 4.82°, 5.46°, 6.57°, 7.29°, 9.47°, and approximately 20.06°, corresponding to the (100), (200), (120), (300), (130), (400), (330), and (001) facets, respectively. These findings aligned well with the PXRD results obtained from the simulations in the AA stacking mode but did not match with the PXRD pattern simulated in the AB stacking mode (Figure S3a, blue line, Figure S3b and Figure S5). The Pawley refined lattice parameters of TPE-BD-COF were *a* = *b* = 55.98 Å, *c* = 5.04 Å, and *α* = *β* = *γ* = 90°, with *R*_wp_ = 5.17% and *R*_p_ = 3.24% as refinement results.

Fourier transform infrared spectroscopy (FT-IR) and solid-state ^13^C cross-polarized magic angle spinning nuclear magnetic resonance (^13^C CP/MAS NMR) spectroscopy verified the existence of TPE-BSD-COF and TPE-BD-COF. In their FT-IR spectra, the distinctive vibrational peak of -CHO at 1696 cm^−1^ was noticeably diminished after the condensation reaction. Meanwhile, TPE-BSD-COF exhibited a prominent stretching signal at 1626 cm^−1^, which can be attributed to the C=N moiety, indicating the successful formation of the imine bonds (Figure 2c). A similar phenomenon was also observed by TPE-BD-COF (Figure S3c). Furthermore, based on Figure 2d and Figure S3d, the chemical shifts at 164.18 ppm (TPE-BSD-COF) and 160.20 ppm (TPE-BD-COF) are attributed to the imine carbons. Additionally, the peaks of TPE-BSD-COF at 158.67 and 151.12 ppm, as well as the peaks of TPE-BD-COF at 153.30 and 148.96 ppm, are specifically associated with benzoselenadiazole and benzothiadiazole units. The broader peaks observed at 110.25–140.90 ppm in both COFs are related to the carbons present in the benzene ring. Measurements using X-ray photoelectron spectroscopy (XPS) were then performed to obtain insights into the elemental composition and valence states of the COFs. Examination of the XPS survey spectra of TPE-BSD-COF and TPE-BD-COF revealed the presence of Se and S elements, in addition to C, N, and O elements (Figure 2e and Figure S3e). This confirms the successful incorporation of benzoselenadiazole and benzothiadiazole into the frameworks of TPE-BSD-COF and TPE-BD-COF, respectively. In the case of TPE-BSD-COF, the Se 3d spectra showed binding energies around 57.5 and 58.3 eV, which correspond to the Se 3d 5/2 and Se 3d 3/2 peaks of the benzoselenadiazole unit (Figure 2f) [42]. The S 2p spectra in TPE-BD-COF displayed S binding energies at approximately 165.8 and 167.0 eV, which can be attributed to the S 2p 3/2 and S 2p 1/2 of the benzothiadiazole unit, respectively (Figure S3f) [43].

**Figure 3:**
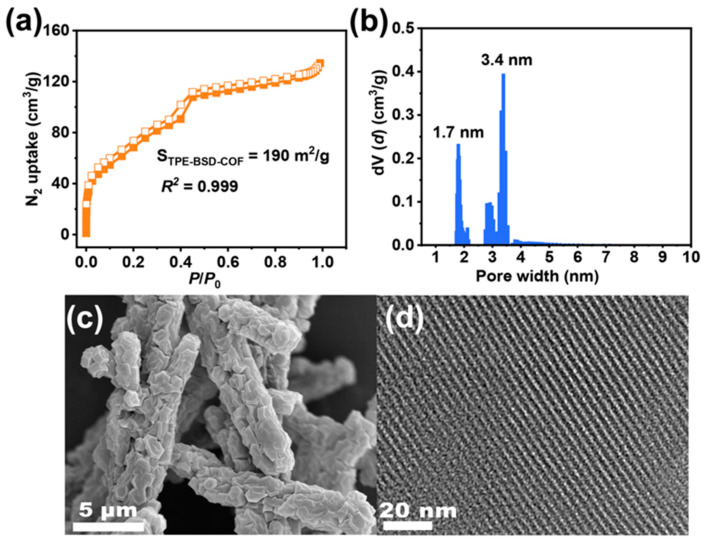
Nitrogen adsorption isotherm (**a**), pore size distribution (**b**), HR-SEM image (**c**), and HR-TEM image (**d**) of TPE-BSD-COF.

Figure 3a and Figure S6a illustrate the Brunauer–Emmett–Teller (BET) surface areas of TPE-BSD-COF and TPE-BD-COF as 190 and 1040 m^2^ g^−1^, respectively. Both COFs display typical type IV isotherms, indicating their porous nature. The pore widths of TPE-BSD-COF, analyzed using nonlocal density functional (NLDFT), are centered at 1.7 and 3.4 nm for TPE-BSD-COF (Figure 3b) and 1.7 and 4.1 nm (Figure S6b) for TPE-BD-COF, respectively. These findings align with the simulated AA layer structure.

High-resolution scanning electron microscopy (HR-SEM) and high-resolution transmission electron microscopy (HR-TEM) were used to examine the morphology of the two COFs. As shown in Figure 3c and Figure S6c, both COFs exhibit a rod-like morphology with a diameter of about 2 μm. HR-TEM images of TPE-BSD-COF and TPE-BD-COF showed clear lattice fringes, indicating long-range ordered channels, further illustrating their high crystallinity (Figure 3d and Figure S6d). The thermal stability of the two COFs was assessed through thermogravimetric analysis (TGA). Both COFs demonstrated exceptional thermal stability above 500 °C with a weight loss of less than 5%, as illustrated in Figure S7.

### 3.2. Photophysical/Electrochemical Properties

**Figure 4:**
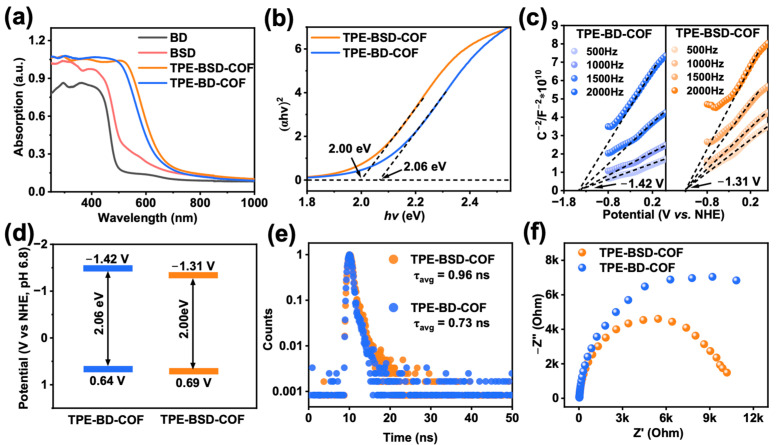
UV–vis spectra of BD, BSD, TPE-BD-COF, and TPE-BSD-COF (**a**). The optical band gap of TPE-BD-COF and TPE-BSD-COF was identified using the Tauc plot (**b**). Mott−Schottky plots of TPE-BD-COF and TPE-BSD-COF (**c**). Energy band structures of TPE-BD-COF and TPE-BSD-COF (**d**). Time-resolved PL spectra of TPE-BD-COF and TPE-BSD-COF (**e**). EIS Nyquist plots of TPE-BD-COF and TPE-BSD-COF (**f**).

Before investigating the suitability of TPE-BSD-COF and TPE-BD-COF for photocatalytic organic conversion, we compared their photoelectric properties. The optical absorption properties were analyzed using solid-state UV–visible diffuse reflectance spectroscopy. Figure 4a illustrates that the light absorption ranges of the two COFs are notably broader than those of the monomers, extending beyond 525 nm. This expansion is primarily attributed to the conjugated system of the COF material. Specifically, the light absorption range of TPE-BSD-COF is red-shifted by approximately 20 nm compared to TPE-BD-COF, suggesting a wider absorption range. The optical bandgaps of both COFs were calculated using Tauc diagrams, resulting in bandgaps of 2.00 eV for TPE-BSD-COF and 2.06 eV for TPE-BD-COF (Figure 4b). Mott-Schottky measurements determined the conduction band minimum (CBM) of −1.31 V vs. NHE for TPE-BSD-COF and −1.42 V vs. NHE for TPE-BD-COF, as shown in Figure 4c. According to the formula Eg = EVB − ECB, the valence band maxima (VBM) of TPE-BSD-COF and TPE-BD-COF are estimated to be 0.69 and 0.64 V (vs. NHE, ignoring exciton binding energy in the optical bandgap), respectively [44]. The band structure arrangement is illustrated in Figure 4d. Time-resolved fluorescence decay spectroscopy was performed on TPE-BSD-COF and TPE-BD-COF. The data, depicted in Figure 4e and Table S3, demonstrated an average lifetime of 0.96 ns for TPE-BSD-COF and 0.73 ns for TPE-BD-COF. This increased lifetime observed in TPE-BSD-COF implies better charge carrier separation compared to TPE-BD-COF, which indicates the superior photocatalytic performance of the former [45]. In addition, the EIS Nyquist diagram (Figure 4f) demonstrates that the semicircle diameter of TPE-BSD-COF is reduced compared to TPE-BD-COF. This observation implies that photogenerated charge carriers in TPE-BSD-COF are transported and separated more efficiently, resulting in a more proficient limitation of electron–hole recombination [46].

### 3.3. Photocatalytic Performance and Possible Mechanisms

**Figure 5:**
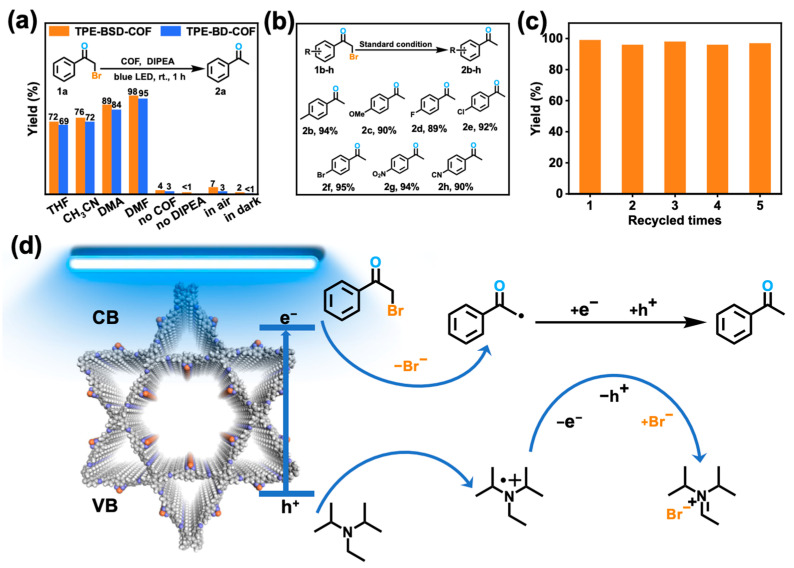
Variations in reaction conditions: **1a** (0.4 mmol), DIPEA (120 mg, 0.88 mmol), COFs (6 mg), solvent (1 mL), and 18 W blue LED in an N_2_ environment at room temperature for 1 h. Yield based on **1a** (**a**). The substrate range for *α*-bromoacetophenone analogues (**b**). Recycling of TPE-BSD-COF in photocatalytic debromination of **1a** (**c**). Schematic diagram of photocatalytic debromination reaction mechanism (**d**).

By combining the optical band gap, energy level arrangement, and electrochemical impedance spectra (EIS) results of the two COFs, it can be inferred that TPE-BSD-COF and TPE-BD-COF possess photocatalytic activity for the reductive dehalogenation of phenacyl bromide derivatives. Optimizing the reaction conditions involved using *α*-bromoacetophenone (1a) as the model substrate, TPE-BSD-COF and TPE-BD-COF as the photocatalyst, and *N,N*-diisopropylethylamine (DIPEA) as the deacid agent, electron donor, and source of hydrogen (Figure 5a). The yield of acetophenone (2a) was measured to be 98% for the TPE-BSD-COF and 95% for the TPE-BD-COF catalyst after 1 h of irradiation with a blue LED lamp (460–465 nm, 25 W) using DMF as the solvent. In contrast, the yield decreased when acetonitrile (CH_3_CN), tetrahydrofuran (THF), and dimethylacetamide (DMA) were used as solvents, respectively. The high yield obtained in DMF can be attributed to its high polarity, which improves the dispersion of COFs in the reaction system, thus allowing more active sites to be exposed and resulting in higher photocatalytic yield. In the absence of COFs, DIPEA, or light setups, only trace amounts of products were detected, demonstrating their combined influence on the photocatalytic reaction. Specifically, blank experiments conducted without COFs yielded only trace amounts of products, attributable to the absence of a light trapping and absorption platform that is characteristic of highly conjugated COFs. This deficiency is unfavorable for the generation of photogenerated charges. Consequently, COFs are essential for effective photocatalysis (Tables S4 and S5). Seven additional compounds were examined to evaluate the viability of the debromination method for various *α*-bromoacetophenone derivatives (Figure 5b). The results showed that substituents that either donate or accept electrons did not significantly affect the rate of the debromination reaction, and the resulting yields of products (2b–2h) stayed at high levels. By reducing the photocatalytic reaction time to 0.5 h, the yield of 2a–h decreased primarily due to an incomplete reaction. Additionally, the substituents did not significantly impact the yield of the debromination reaction, highlighting the versatility of TPE-BSD-COF as photocatalysts for dehalogenation processes (Table S6). Cycling experiments were conducted to assess the structural stability of TPE-BSD-COF after photocatalysis. The results showed that the TPE-BSD-COF catalyst was able to maintain its structural integrity after undergoing repeated filtration, washing, and drying processes. Additionally, the yield of the products remained consistent even after five cycles of use, indicating that the TPE-BSD-COF catalyst is durable and can be easily separated and reused multiple times without a significant decrease in performance (Figure 5c). PXRD and SEM analyses further validated the preservation of crystallinity and structural integrity of TPE-BSD-COF after five rounds of photocatalytic cycling, indicating that TPE-BSD-COF exhibits remarkable stability (Figure S8).

Drawing from existing studies [47], a potential mechanism for the debromination process of *α*-bromoacetophenone was suggested, illustrated in Figure 5d. Under the illumination of LED light, the photogenerated electrons are transferred from the conduction band of TPE-BSD-COF or TPE-BD-COF to *α*-bromoacetophenone, forming an *α*-carbonyl radical and a bromide anion. The *α*-carbonyl radical then abstracts an H^+^ and electrons from DIPEA, leading to the production of acetophenone. In this reaction, DIPEA serves both as a hydrogen source and a deacidifying reagent, forming a by-product.

## 4. Conclusions

In summary, two benzodiazole-based heteropore COFs, i.e., benzoselenadiazole-containing TPE-BSD-COF and benzothiadiazole-containing TPE-BD-COF, have been successfully designed and constructed by imine condensation reaction under solvothermal conditions. Both TPE-BSD-COF and TPE-BD-COF exhibit excellent photoelectric characteristics such as an extensive light absorption spectrum, reduced optical bandgap, minimal EIS, and long fluorescence lifetime. These traits indicate that the two COFs possess strong charge-transferring and separation abilities, leading to improved performance in photocatalytic debromination ability for *α*-bromoacetophenone. Among them, the yield of photocatalytic dehalogenation catalyzed by TPE-BSD-COF reached 98% within one hour. This study not only broadens the range of COFs but also highlights their potential as photocatalysts for the photocatalytic dehalogenation of phenylacetylbromide derivatives, which is of great informative importance.

## Data Availability

The data presented in this study are available on request from the corresponding author.

## Supplementary Materials

The following supporting information can be downloaded at: https://www.mdpi.com/article/10.3390/polym16182578/s1, Figure S1: Synthesis route of TPE-BSD-COF; Figure S2: Synthesis route of TPE-BD-COF; Figure S3: The experimental and simulated PXRD patterns of TPE-BD-COF (a). Structural model of TPE-BD-COF (b). FT-IR spectra of TPE-BD-COF and the monomers (c). The solid-state ^13^C CP/MAS NMR spectra of TPE-BD-COF (d). Survey-scan XPS spectrum of TPE-BD-COF (e). High-resolution S 2p XPS spectrum of TPE-BD-COF (f); Figure S4: The experimental (orange) and simulated (black) PXRD patterns of TPE-BSD-COF; Figure S5: The experimental (bule) and simulated (black) PXRD patterns of TPE-BD-COF; Figure S6: Nitrogen adsorption isotherm (a), pore size distribution (b), HR-SEM image (c) and HR-TEM image (d) of TPE-BD-COF; Figure S7: TGA curves of TPE-BSD-COF and TPE-BD-COF; Figure S8: PXRD pattern of the recycled TPE-BSD-COF (a) and SEM image of the recycled TPE-BSD-COF (b); Figure S9: ^1^H NMR spectrum (400 MHz, CDCl_3_) δ 8.06 (d, *J* = 8.8 Hz, 8H), 7.19 (d, *J* = 8.8 Hz, 8H); Figure S10: ^1^H NMR spectrum (400 MHz, DMSO-*d*6) δ 6.57 (d, *J* = 8.4 Hz, 4H), 6.26 (d, *J* = 8.4 Hz, 4H), 4.84 (s, 4H); Figure S11: ^1^H NMR spectrum (400 MHz, CDCl_3_) δ 10.13 (s, 2H), 8.18 (d, *J* = 8.4 Hz, 4H), 8.08 (d, *J* = 8.4 Hz, 4H), 7.91 (s, 2H); Figure S12: ^1^H NMR spectrum (400 MHz, DMSO-*d*6) δ 10.11 (s, 2H), 8.19 (d, *J* = 8.0 Hz, 4H), 8.07 (d, *J* = 8.1 Hz, 4H), 7.91 (s, 2H); Figure S13: ^1^H NMR spectrum (400 MHz, CDCl_3_) δ 7.96–7.90 (m, 2H), 7.56–7.49 (m, 1H), 7.47–7.40 (m, 2H), 2.57 (s, 3H); Figure S14: ^13^C NMR spectrum (100 MHz, CDCl_3_) δ 198.15, 137.12, 133.11, 128.58, 128.31, 77.47, 77.15, 76.83, 26.58; Figure S15: ^1^H NMR spectrum (400 MHz, CDCl_3_) δ 7.83 (d, *J* = 8.4 Hz, 2H), 7.22 (d, *J* = 7.8 Hz, 2H), 2.54 (s, 3H), 2.37 (s, 3H); Figure S16: ^13^C NMR spectrum (100 MHz, CDCl_3_) δ 197.82, 143.87, 134.71, 129.25, 128.44, 77.49, 77.17, 76.85, 26.49, 21.60; Figure S17: ^1^H NMR spectrum (400 MHz, CDCl_3_) δ 7.90 (d, *J* = 8.8 Hz, 2H), 6.89 (d, *J* = 8.8 Hz, 2H), 3.82 (s, 3H), 2.51 (s, 3H); Figure S18: ^13^C NMR spectrum (100 MHz, CDCl_3_) δ 196.77, 163.51, 130.59, 130.33, 113.69, 77.46, 77.15, 76.83, 55.45, 26.32; Figure S19: ^1^H NMR spectrum (400 MHz, CDCl_3_) δ 7.92 (dd, *J* = 8.8 Hz, 2H), 7.06 (t, *J* = 8.8 Hz, 2H), 2.52 (s, 3H); Figure S20: ^13^C NMR spectrum (100 MHz, CDCl_3_) δ 196.49, 167.07, 164.54, 133.68, 133.65, 131.05, 130.95, 115.78, 115.56, 77.55, 77.23, 76.91, 26.51; Figure S21: ^1^H NMR spectrum (400 MHz, CDCl_3_) δ 7.85 (d, *J* = 8.4 Hz, 2H), 7.38 (d, *J* = 8.4 Hz, 2H), 2.54 (s, 3H); Figure S22: ^13^C NMR spectrum (100 MHz, CDCl_3_) δ 196.80, 139.53, 135.43, 129.74, 128.88, 77.46, 77.14, 76.82, 26.54; Figure S23: ^1^H NMR spectrum (400 MHz, CDCl_3_) δ 7.76 (d, *J* = 8.6 Hz, 2H), 7.54 (d, *J* = 8.6 Hz, 2H), 2.53 (s, 3H); Figure S24: ^13^C NMR spectrum (100 MHz, CDCl_3_) δ 196.93, 135.78, 131.84, 129.82, 128.24, 77.45, 77.13, 76.82, 26.50; Figure S25: ^1^H NMR spectrum (400 MHz, CDCl_3_) δ 8.27 (d, *J* = 8.8 Hz, 2H), 8.08 (d, *J* = 8.8 Hz, 2H), 2.65 (s, 3H); Figure S26: ^13^C NMR spectrum (100 MHz, CDCl_3_) δ 196.42, 150.38, 141.44, 129.37, 123.88, 77.47, 77.15, 76.84, 27.01; Figure S27: ^1^H NMR spectrum (400 MHz, CDCl_3_) δ 8.06 (d, *J* = 8.4 Hz, 1H), 7.79 (d, *J* = 8.6 Hz, 1H), 2.66 (s, 2H); Figure S28: ^13^C NMR spectrum (100 MHz, CDCl_3_) δ 196.62, 139.94, 132.54, 128.73, 117.97 pm, 116.36, 77.49, 77.17, 76.85, 26. Table S1: Fractional atomic coordinates of the TPE-BSD-COF unit cell; Table S2: Fractional atomic coordinates of the TPE-BD-COF unit cell; Table S3: The PL decay lifetimes of the studied COFs; Table S4: Photocatalytic reductive dehalogenation of TPE-BSD-COF.^a^ Table S5: Photocatalytic reductive dehalogenation of TPE-BD-COF.^a^ Table S6: Photocatalytic dehalogenation reactions catalyzed by TPE-BSD-COF.
