# Supplementary material for: Benzodiazole-Based Covalent Organic Frameworks for Enhanced Photocatalytic Dehalogenation of Phenacyl Bromide Derivatives
Source: Polymers (Basel). 2024 Sep 12;16(18):2578. doi: 10.3390/polym16182578 (PMC11435578; doi:10.3390/polym16182578)
Supplement: Supplementary file 1 [file polymers-16-02578-s001.zip › polymers-3146147-supplementary.pdf]

polymers

MDPI

Supplementary Material

Benzodiazole-based covalent organic frameworks for enhanced photocatalytic dehalogenation of phenacyl bromide derivatives

Ming Wang [1], Jiaying Qian [1], Shenglin Wang [1], Zhongliang Wen [1,*], Songtao Xiao [2,*], Hui Hu [1], and Yanan Gao [1,*]

[1] Key Laboratory of Ministry of Education for Advanced Materials in Tropical Island Resources, Hainan University, No 58, Renmin Avenue, Haikou 570228, China; ygao@hainanu.edu.cn

[2] China Institute of Atomic Energy. Beijing 102413, China; xiao200112@163.com

Section A. Materials and Characterization

Electrochemical measurements

Electrochemical impedance spectroscopy (EIS), Photocurrent, and Mott-Schottky plots of TPE-BD-COF and TPE-BSD-COF were performed on an electrochemical workstation Ivium-CompactStat.h via a standard three-electrode system in 0.2 M Na_2SO_4 (pH = 6.8) solution, which contains a working electrode, a platinum plate as counter electrode, and a saturated Ag/AgCl electrode as a reference electrode. The catalyst was dispersed into a solution of 20 μL 5 wt % Nafion and 1 mL EtOH. Then the resulting mixture (30 μL) was deposited onto the surface of the ITO conductive glass and left in the air for drying to prepare the working electrode. The electrochemical impedance measurements were carried out at open-circuit voltage with AC amplitude of 20 mV in the frequency range of 0.01 Hz to 10^5 Hz. Mott-Schottky plots were recorded at frequencies of 500 and 1000 Hz, respectively. The applied potentials vs. Ag/AgCl were converted to NHE potentials using the following equation:

$$E_{NHE} = E(Ag/AgCl) + E^{\theta}_{(Ag/AgCl)}\ (E^{\theta}_{(Ag/AgCl)} = 0.199\ V), \quad (1)$$

General Procedure of Photocatalytic Reductive Dehalogenation of 2-Bromoacetophenone.

A 10 mL Pyrex glass tube equipped with a stir bar was charged with 2-bromoacetophenone (1a) (0.4 mmol), *N*,*N*-diisopropylethylamine (DIPEA) (0.4 mmol), COF (3 mg), and DMF (1.0 mL), and the reaction mixture was irradiated in the photoreactor (18 W blue LEDs, 460–465 nm) with a cooling fan for 1 h under an N_2 atmosphere. After the reaction ended, the solid was collected by filtration, and the filtrate was concentrated under reduced pressure. The residue was purified by flash chromatography on silica gel with PE/EtOAc as the eluent to afford the products. Liquid-state ^{1}H NMR and ^{13}C NMR spectra were recorded at ambient temperature using a Bruker III HD spectrometer operating at 600 MHz, (^{1}H NMR) and 151 MHz, (^{13}C NMR), and chemical shift (δ) values were reported in parts per million (ppm) relative to the residual solvent peaks ($CDCl_3$ δ 7.26 ppm for ^{1}H NMR and CDCl3 δ 77.16 ppm for ^{13}C NMR). Peaks were assigned as s = singlet, d = doublet, and the coupling constants were reported in Hz. The yield of acetophenone and its derivatives was analyzed by gas chromatography (GC).

Computational details

The lattice models were optimized using the Forcite module and corresponding diffraction patterns were simulated in the Reflex module.

Section B. Synthetic Procedures

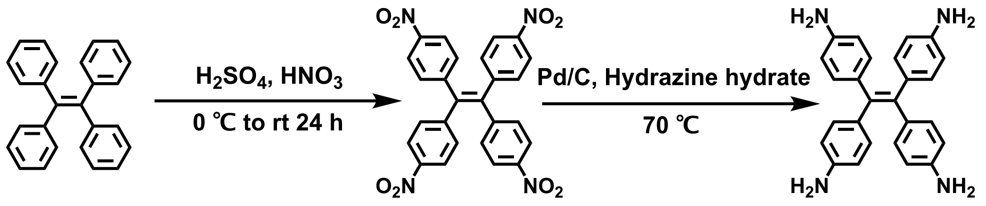

A B C

Synthesis of 1,1,2,2-tetrakis(4-nitrophenyl)ethene (B)

A (2.0 g, 6 mmol) was dissolved in a mixture of sulfuric acid and concentrated nitric acid (30 mL/60 mL). 30 mL concentrated nitric acid was then added slowly to the reaction mixture, and the mixture was stirred for 24 h at room temperature. The reaction mixture was poured into 300 mL of ice water to obtain a light-yellow precipitate. After filtration, the filtrate was concentrated to afford crude product, which was recrystallized from 1,4-dioxane to give B as a light-yellow solid (1.54 g, 50%).

Synthesis of 4,4′,4′′,4′′′-(ethene-1,1,2,2-tetrayl)tetraaniline (C)

A 250 mL flame-dried round bottom flask was charged with B (2.5 g, 5 mmol), Pd/C (10%, 450 mg), hydrazine hydrate (23 mL, 300 mmol), and ethanol (120 mL). The mixture was degassed for three times, and then the reaction mixture was stirred at 70 °C for 12 h. Then the mixture was filtered and the filtrate was concentrated. After addition of dichloromethane (200 mL), the organic phase was washed with water and dried over anhydrous Na_2SO_4. After filtration, the filtrate was concentrated to give crude product, which was purified by column chromatography on silica gel (DCM/EtOAc, 5:1 v/v) to afford C as a yellow solid (1.1 g, 55%). ^{1}H NMR (400 MHz, DMSO-*d6*) δ 6.57 ppm (d, *J* = 8.4 Hz, 4H), 6.26 ppm (d, *J* = 8.4 Hz, 4H) 4.84 ppm (s, 4H).

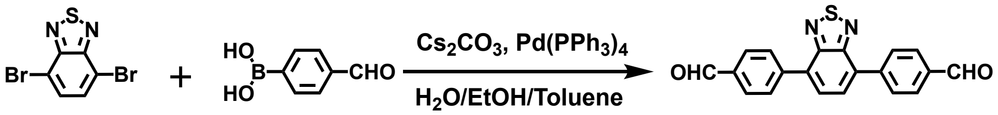

A B C

Synthesis of 4,4′-(Benzothiadiazole-4,7-diyl)dibenzaldehyde (BD)

To a Cs_2CO_3/H_2O solution (6.41 g/10 mL) in a 200 mL two-necked flask was added distilled EtOH/toluene (20 mL /80 mL), A (1.18 g, 4 mmol), B (1.51 g, 10 mmol), and $Pd(PPh_3)_4$ (0.23 g, 0.2 mmol). The reaction mixture was degassed three times. After reflux in N_2 over 36 h, the mixture was poured into water and extracted with chloroform three times and the organic solvents were removed by rotary evaporator. The resulting greenish solid was purified through silica gel column chromatography using dichloromethane as an eluent to afford C as a green powder (1.35 g, 97%). ^{1}H NMR (400 MHz, $CDCl_3$) δ 10.13 ppm (s, 2H), 8.18 ppm (d, *J* = 8.4 Hz, 4H), 8.08 ppm (d, *J* = 8.4 Hz, 4H), 7.91 ppm (s, 2H).

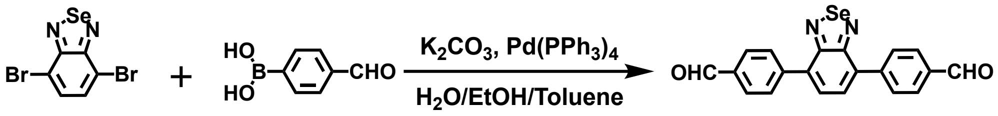

A B C

Synthesis of 4,4′-(benzoselenadiazole-4,7-diyl)dibenzaldehyde (BSD)

To a K_2CO_3/H_2O solution (8.20 g/10 mL) in a 200 mL two-necked flask was added distilled EtOH/toluene (20 mL /80 mL), A (1.23 g, 4 mmol), B (1.50 g, 10 mmol), and $Pd(PPh_3)_4$ (0.23 g, 0.2 mmol). The reaction mixture was degassed three times. After reflux in N_2 over 48 h, the mixture was poured into water and extracted with chloroform three

times and the organic solvents were removed by rotary evaporator. The resulting greenish solid was purified through silica gel column chromatography using dichloromethane as an eluent to afford C as a yellow powder (1.2 g, 75%). ^{1}H NMR (400 MHz, DMSO-*d6*) δ 10.11 ppm (s, 2H), 8.19 ppm (d, *J* = 8.0 Hz, 4H), 8.07 ppm (d, *J* = 8.1 Hz, 4H), 7.91 ppm (s, 2H).

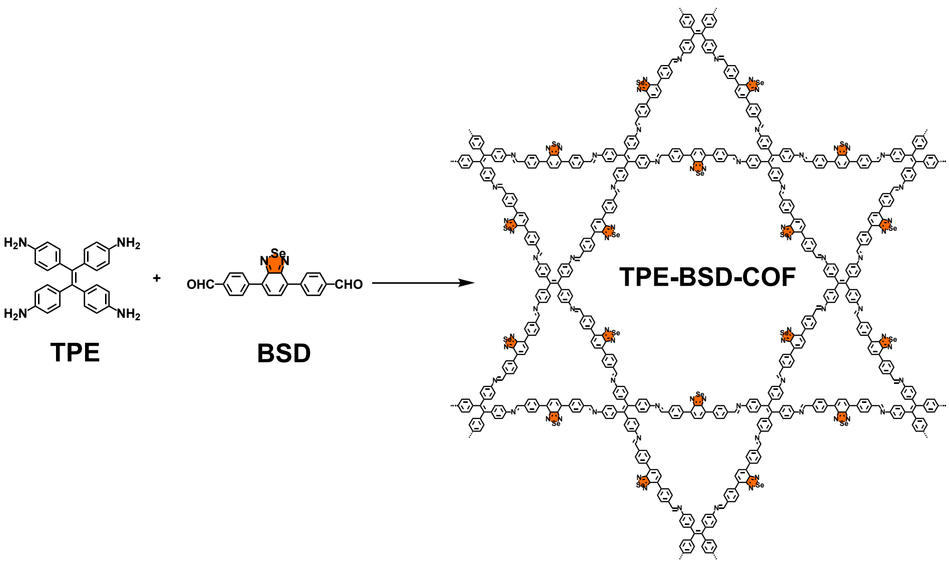

Figure S1. Synthesis route of TPE-BSD-COF.

Synthesis of TPE-BSD-COF

TPE (9.8 mg, 0.025 mmol) and BSD (20.0 mg, 0.05 mmol) were taken in a seal tube and a mixture of *o*-DCB/*n*-butanol (2 mL, v/v 1/1) and 3M acetic acid (0.1 mL) was added into the tube. The tube was sonicated for five minutes and degassed via a freeze-pump-thaw process. Then, the tube was sealed and allowed the reaction to proceed at 120 ºC for 3 days. The precipitate was collected, washed with THF, and dried under vacuum to yield a yellow COF powder in 79% isolated yield.

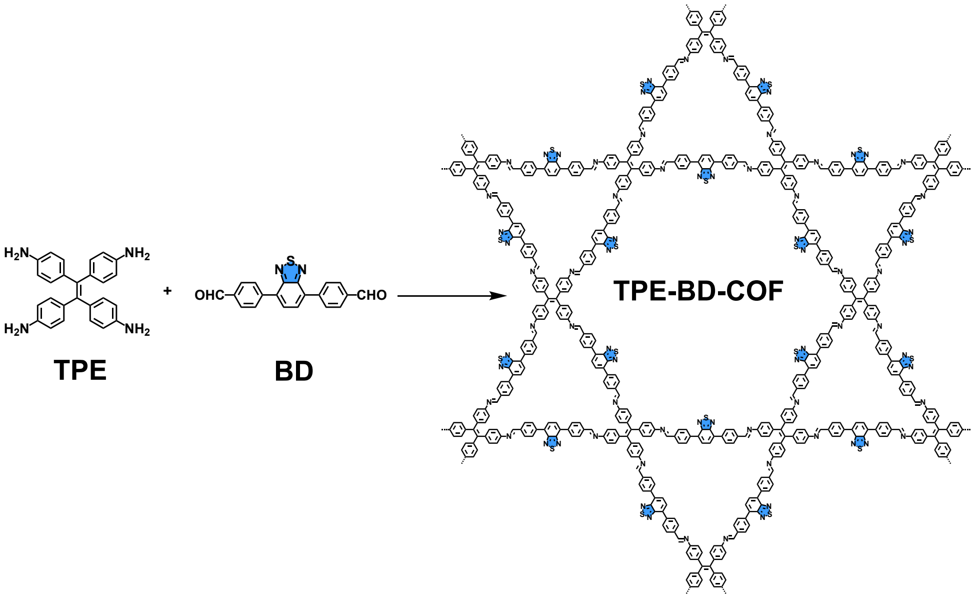

Figure S2. Synthesis route of TPE-BD-COF.

Synthesis of TPE-BD-COF

TPE (9.8 mg, 0.025 mmol) and BD (17.8 mg, 0.05 mmol) were taken in a seal tube and a mixture of *o*-DCB/*n*-butanol (2 mL, v/v 1/1) and 3M acetic acid (0.1 mL) was added into the tube. The mixture was sonicated for 5 minutes and then degassed via a freeze-pump-thaw process. The tube was sealed and allowed the reaction proceed at 120 ºC for 3 days. The precipitate was collected, washed with THF, and dried under vacuum to yield a yellow COF powder in 82% isolated yield.

Section C. Results and Discussion

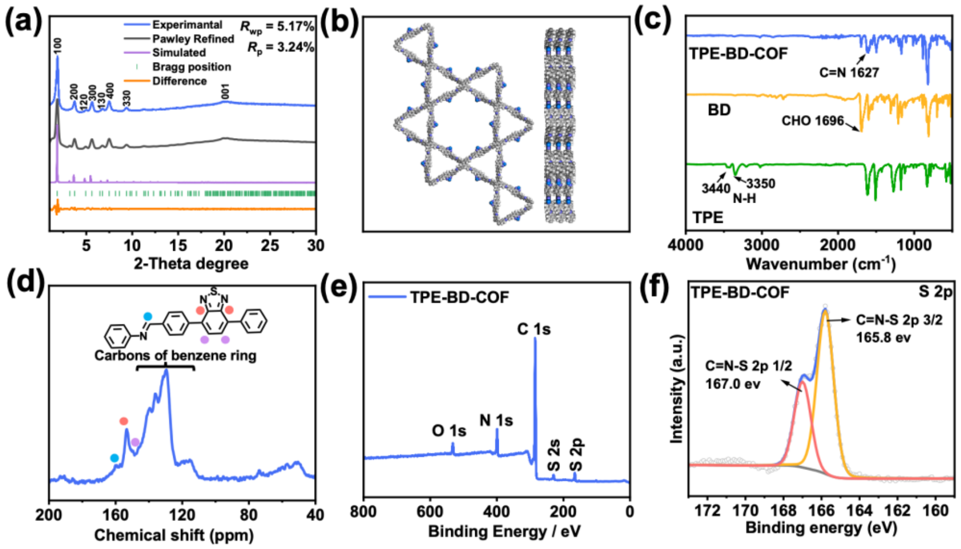

Figure S3. The experimental and simulated PXRD patterns of TPE-BD-COF (a). Structural model of TPE-BD-COF (b). FT-IR spectra of TPE-BD-COF and the monomers (c). The solid-state ^{13}C CP/MAS NMR spectra of TPE-BD-COF (d). Survey-scan XPS spectrum of TPE-BD-COF (e). High-resolution S 2p XPS spectrum of TPE-BD-COF (f).

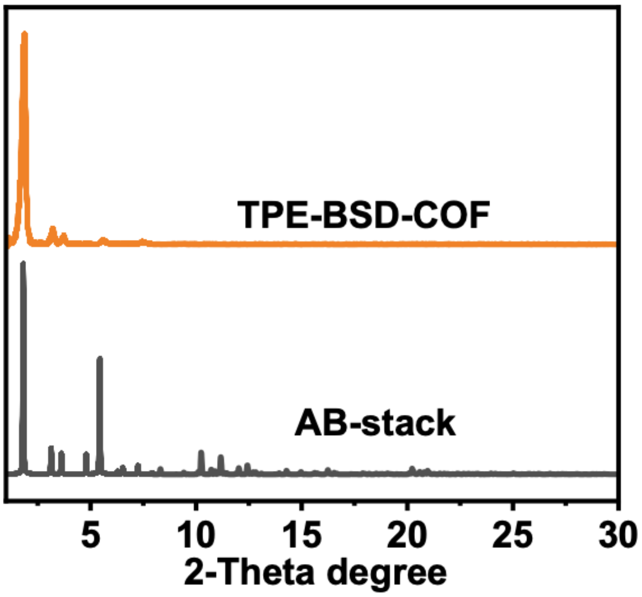

Figure S4. The experimental (orange) and simulated (black) PXRD patterns of TPE-BSD-COF.

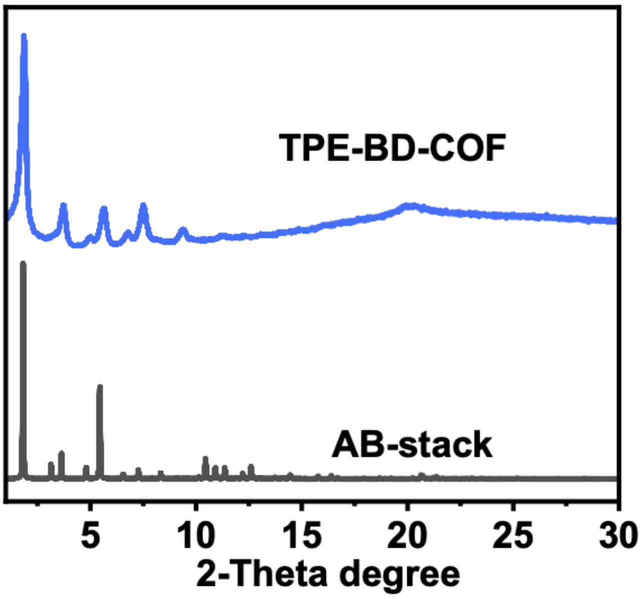

Figure S5. The experimental (bule) and simulated (black) PXRD patterns of TPE-BD-COF.

Table S1. Fractional atomic coordinates of the TPE-BSD-COF unit cell.

TPE-BSD-COF			
$a = b = 55.9849$ Å, $c = 5.0417$ Å			
Hexagonal, *P*6			
$\alpha = \beta = 90°$, $\gamma = 120°$			
Atom	X	y	z
C1	0.45989	0.48504	0.41224
C2	0.44004	0.4663	0.23353
C3	0.41407	0.464	0.21151
C4	0.45258	0.5008	0.57322
C5	0.47142	0.43919	0.58825
C6	0.4701	0.41365	0.56544
C7	0.50067	0.43059	0.18699
C8	0.50171	0.4559	0.21055
C9	0.36168	0.46629	0.18268
C10	0.33622	0.46802	0.1918
C11	0.31515	0.4532	0.007
C12	0.29109	0.45493	0.00979
C13	0.28775	0.47159	0.19712
C14	0.26271	0.47419	0.196
C15	0.49314	0.37387	0.16949
C16	0.48913	0.34575	0.17772
C17	0.503	0.3383	0.007
C18	0.49983	0.31192	0.00258
C19	0.4825	0.29243	0.18508
C20	0.46848	0.29979	0.36946
C21	0.47181	0.32626	0.36638
C22	0.50309	0.2617	0.19226

C23	0.4536	0.24055	0.19168
C24	0.23609	0.45102	0.19326
N25	0.48223	0.38222	0.34856
C26	0.48785	0.48749	0.42632
C27	0.4876	0.46073	0.41247
C28	0.47954	0.26465	0.19043
C29	0.50044	0.23539	0.19689
C30	0.40724	0.48035	0.37094
C31	0.3088	0.48642	0.38201
C32	0.33278	0.48458	0.38031
N33	0.38119	0.47952	0.35512
C34	0.48461	0.409	0.36468
C35	0.57338	0.50155	0.55353
N36	0.76979	0.57533	0.1945
Se3	0.80427	0.59746	0.1898
N38	0.81046	0.57077	0.18763
H39	0.44468	0.45353	0.10834
H40	0.3997	0.44939	0.07029
H41	0.46714	0.51557	0.70818
H42	0.45973	0.44211	0.74204
H43	0.45759	0.39733	0.70398

Table S2. Fractional atomic coordinates of the TPE-BD-COF unit cell. 111

TPE-BD-COF			
$a = b = 55.9849$ Å, $c = 5.0417$ Å			
Hexagonal, *P*6			
$\alpha = \beta = 90°$, $\gamma = 120°$			
Atom	X	y	z
C1	0.45989	0.48505	0.41241
C2	0.44004	0.46633	0.23366
C3	0.41408	0.46404	0.21163
C4	0.45259	0.50081	0.57347
C5	0.47139	0.43918	0.5883
C6	0.47005	0.41363	0.56556
C7	0.50067	0.43057	0.18751
C8	0.50172	0.45589	0.21095
C9	0.36171	0.46637	0.18284
C10	0.33626	0.46811	0.19181
C11	0.31514	0.45319	0.00772
C12	0.29107	0.4549	0.0105
C13	0.28778	0.47166	0.19703
C14	0.26272	0.47421	0.19597
C15	0.49279	0.37374	0.16785
C16	0.48887	0.34565	0.17655
C17	0.50267	0.33818	0.00872
C18	0.49957	0.31184	0.00391
C19	0.48237	0.29238	0.18468
C20	0.46843	0.29976	0.36963
C21	0.47169	0.32621	0.36618
C22	0.50304	0.26171	0.18969

C23	0.45355	0.24049	0.19398
C24	0.23612	0.45102	0.19564
N25	0.48217	0.38219	0.34883
C26	0.48785	0.48749	0.42641
C27	0.48758	0.46073	0.41261
C28	0.47946	0.26462	0.19024
C29	0.50045	0.23542	0.19441
C30	0.40726	0.48041	0.37107
C31	0.30888	0.48659	0.38113
C32	0.33286	0.48476	0.37952
N33	0.38123	0.4796	0.35513
C34	0.48456	0.40898	0.36489
C35	0.57335	0.50152	0.55373
N36	0.76971	0.57531	0.19946
S37	0.80418	0.59749	0.19719
N38	0.81043	0.57085	0.19245
H39	0.44468	0.45356	0.10843
H40	0.39971	0.44944	0.07037
H41	0.46716	0.51557	0.7085
H42	0.45969	0.44211	0.74197
H43	0.45753	0.39732	0.70401

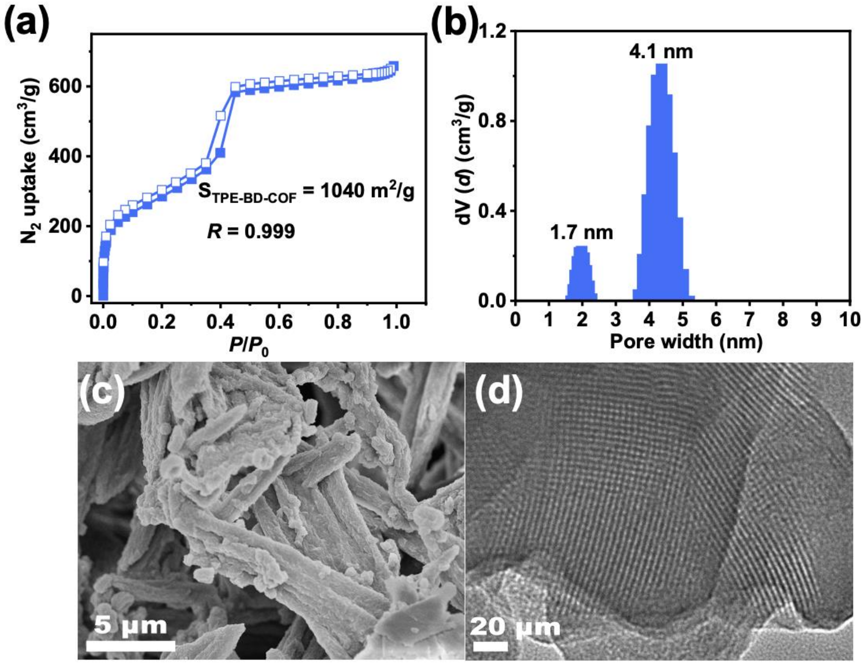

113

Figure S6. Nitrogen adsorption isotherm (a), pore size distribution (b), HR-SEM image (c) and HR-TEM image (d) of TPE-BD-COF. 114 115

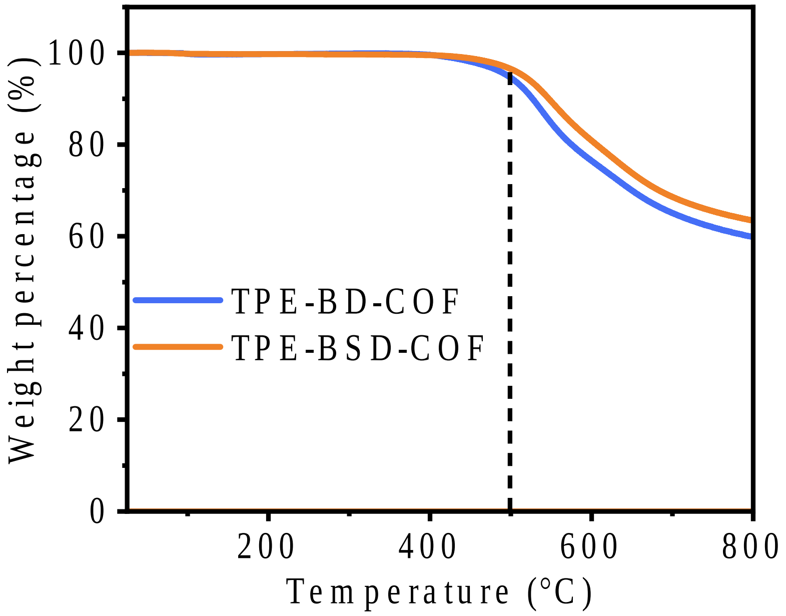

116

Figure S7. TGA curves of TPE-BSD-COF and TPE-BD-COF. 117

Calculation of photoluminescence lifetime

The band-edge emission of COFs decays by the radiative and nonradiative electron-hole recombination processes. The life-time fittings were performed according to a biexponential decay model:

$$y = y_0 + A_1 e^{-(x-x_0)/\tau_1} + A_2 e^{-(x-x_0)/\tau_2} \quad (2)$$

$$\tau = \frac{A_1 {\tau_1}^2 + A_2 {\tau_2}^2}{A_1 \tau_1 + A_2 \tau_2} \quad (3)$$

Table S3. The PL decay lifetimes of the studied COFs.

Sample	A1	τ_1/ns	A2	τ_2/ns	τ/ns
TPE-BSD-COF	1654.01	0.6954	99.1929	2.3142	0.9647
TPE-BD-COF	1645.86	0.5828	83.3410	1.7105	0.7287

where y is intensity, τ, τ_1, and τ_2 are decay times, and A1 and A2 are relative magnitudes.

Polymers **2024**, *16*, x. https://doi.org/10.3390/xxxxx www.mdpi.com/journal/polymers

Table S4. Photocatalytic reductive dehalogenation of TPE-BSD-COF.[a]

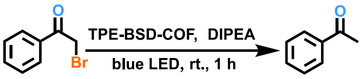

Entry	Photocatalyst	Yield (%)
1	TPE-BSD-COF[b]	72
2	TPE-BSD-COF[c]	76
3	TPE-BSD-COF[d]	98
4	No[e]	4
5	TPE-BSD-COF[f]	0.1
6	TPE-BSD-COF[g]	7
7	TPE-BSD-COF[h]	2

[a]Reaction conditions: 3 mg catalyst, 2-bromoacetophenone (0.40 mmol), *N*,*N*-diisopropylethylamine (DIPEA) (0.40 mmol) and a certain amount of dodecane as the internal standard, irradiation with 18 W blue LEDs, 1 h. [b]Anhydrous THF (1mL) as solvent. [c]Anhydrous CH_3CN (1mL) as solvent. [d]Anhydrous DMF (1mL) as solvent. [e]In the absence of TPE-BSD-COF. [f]No DIPEA. [g]In air. [h]Under dark.

Table S5. Photocatalytic reductive dehalogenation of TPE-BD-COF.[a]

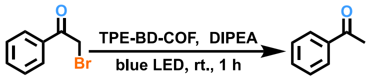

Entry	Photocatalyst	Yield (%)
1	TPE-BD-COF[b]	69
2	TPE-BD-COF[c]	72
3	TPE-BD-COF[d]	95
4	No[e]	3
5	TPE-BD-COF[f]	0.5
6	TPE-BD-COF[g]	3
7	TPE-BD-COF[h]	0.8

[a]Reaction conditions: 3 mg catalyst, 2-bromoacetophenone (0.40 mmol), *N*,*N*-diisopropylethylamine (DIPEA) (0.40 mmol) and a certain amount of dodecane as the internal standard, irradiation with 18 W blue LEDs, 1 h. [b]Anhydrous THF (1mL) as solvent. [c]Anhydrous CH_3CN (1mL) as solvent. [d]Anhydrous DMF (1mL) as solvent. [e]In the absence of TPE-BD-COF. [f]No DIPEA. [g]In air. [h]Under dark.

Table S6. Photocatalytic dehalogenation reactions catalyzed by TPE-BSD-COF in 0.5 h.

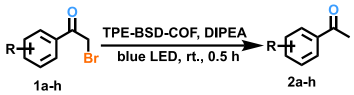

Entry	Substrate	Product	Yield (%) (0.5 h)
1	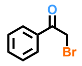	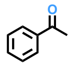	72%
2	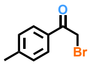	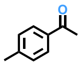	69%
3	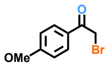	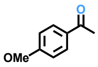	71%
4	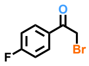	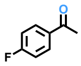	67%
5	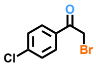	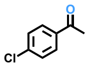	68%
6	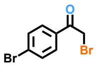	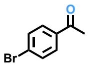	70%
7	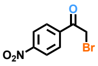	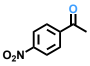	72%
8	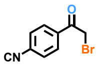	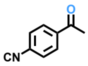	70%

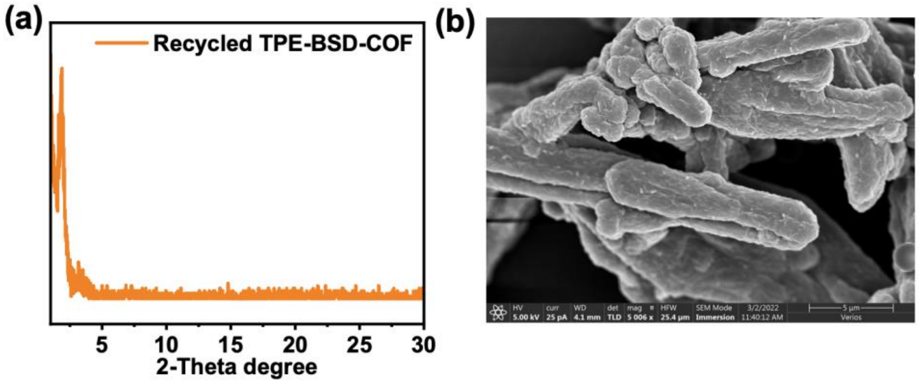

Figure S8. PXRD pattern of the recycled TPE-BSD-COF (a) and SEM image of the recycled TPE-BSD-COF (b).

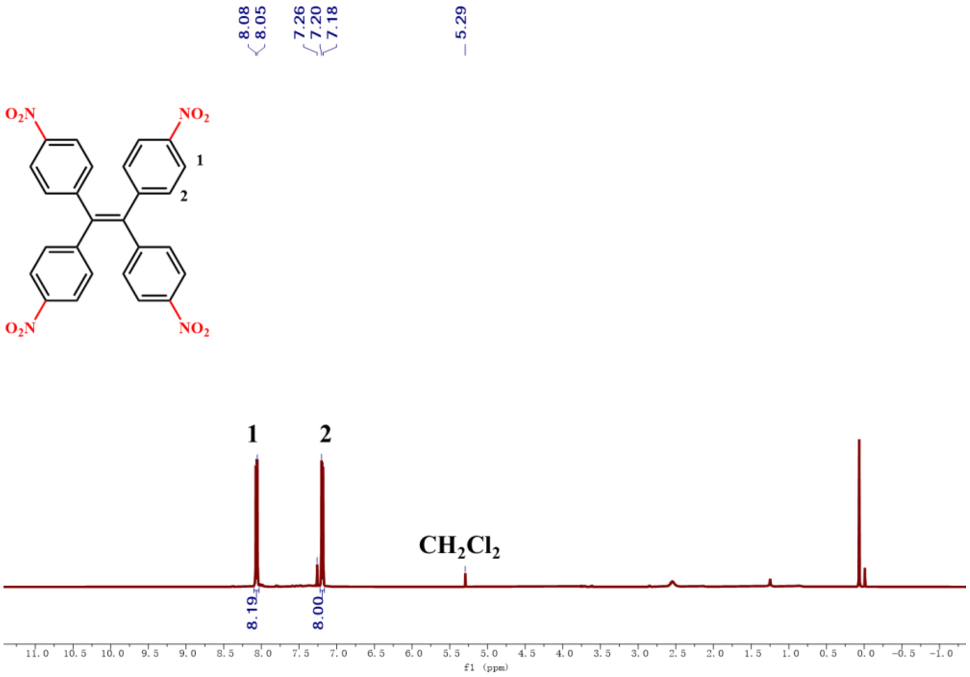

Figure S9. ^{1}H NMR spectrum (400 MHz, $CDCl_3$) δ 8.06 (d, *J* = 8.8 Hz, 8H), 7.19 (d, *J* = 8.8 Hz, 8H).

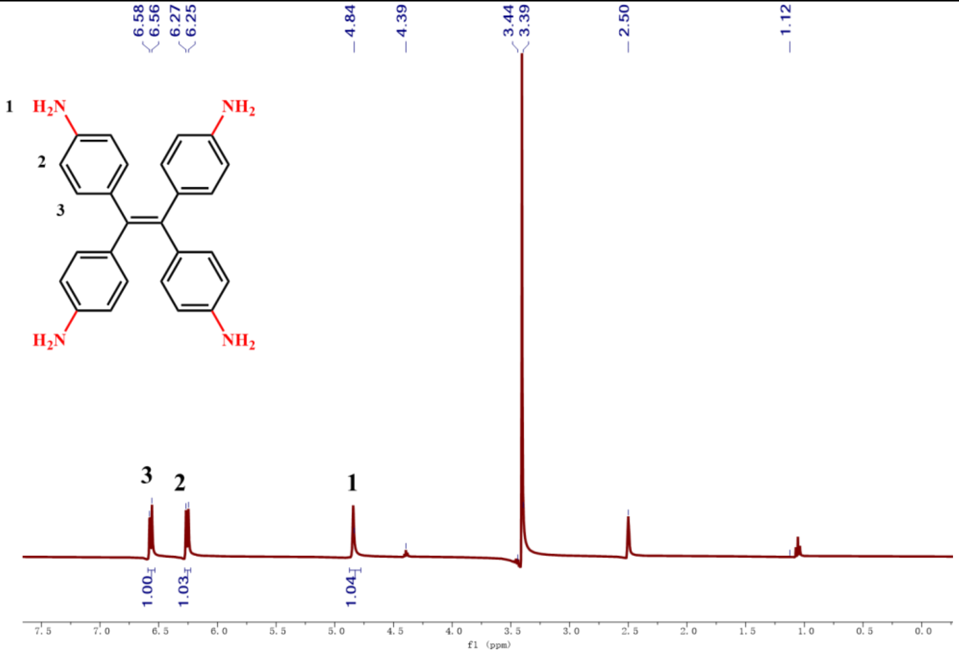

Figure S10. ^{1}H NMR spectrum (400 MHz, DMSO-*d6*) δ 6.57 (d, *J* = 8.4 Hz, 4H), 6.26 (d, *J* = 8.4 Hz, 4H), 4.84 (s, 4H).

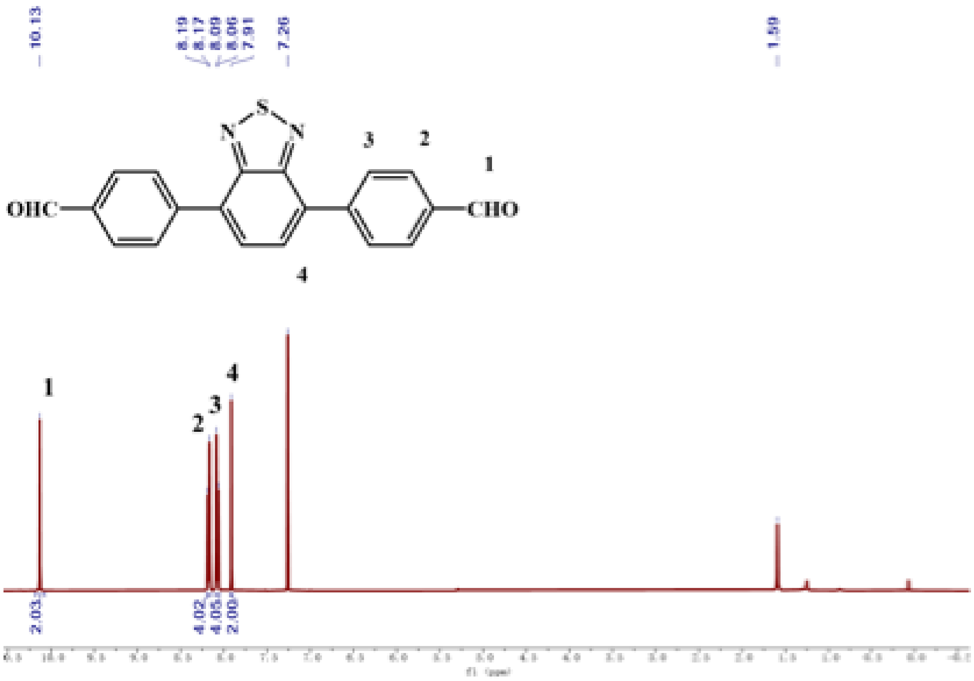

Figure S11. ^{1}H NMR spectrum (400 MHz, $CDCl_3$) δ 10.13 (s, 2H), 8.18 (d, *J* = 8.4 Hz, 4H), 8.08 (d, *J* = 8.4 Hz, 4H), 7.91 (s, 2H).

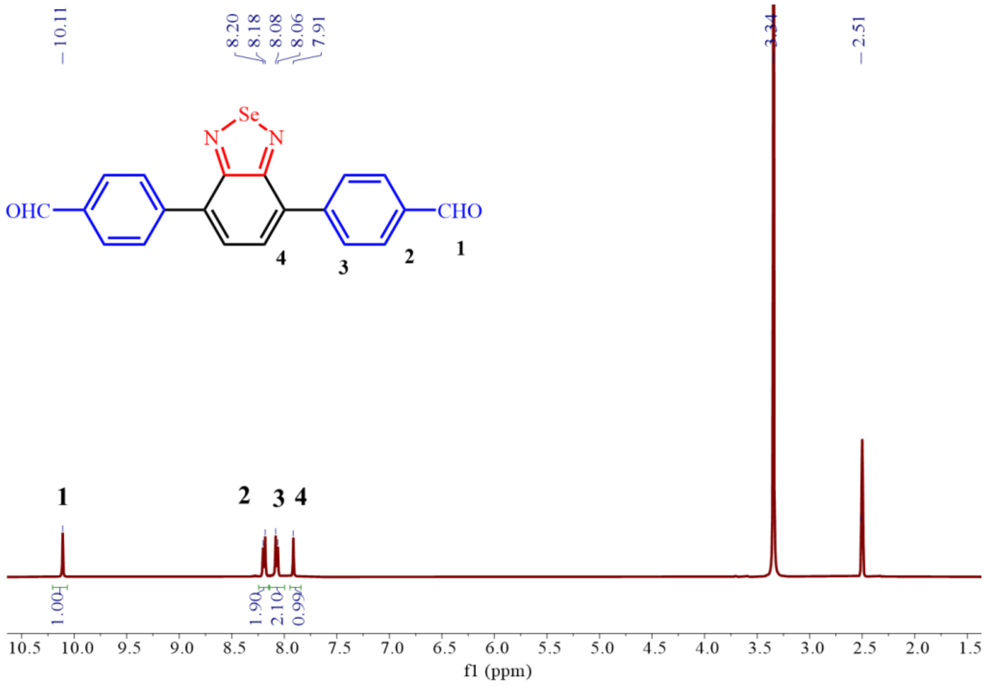

Figure S12. ^1H NMR spectrum (400 MHz, DMSO-*d6*) δ 10.11 (s, 2H), 8.19 (d, *J* = 8.0 Hz, 4H), 8.07 (d, *J* = 8.1 Hz, 4H), 7.91 (s, 2H).

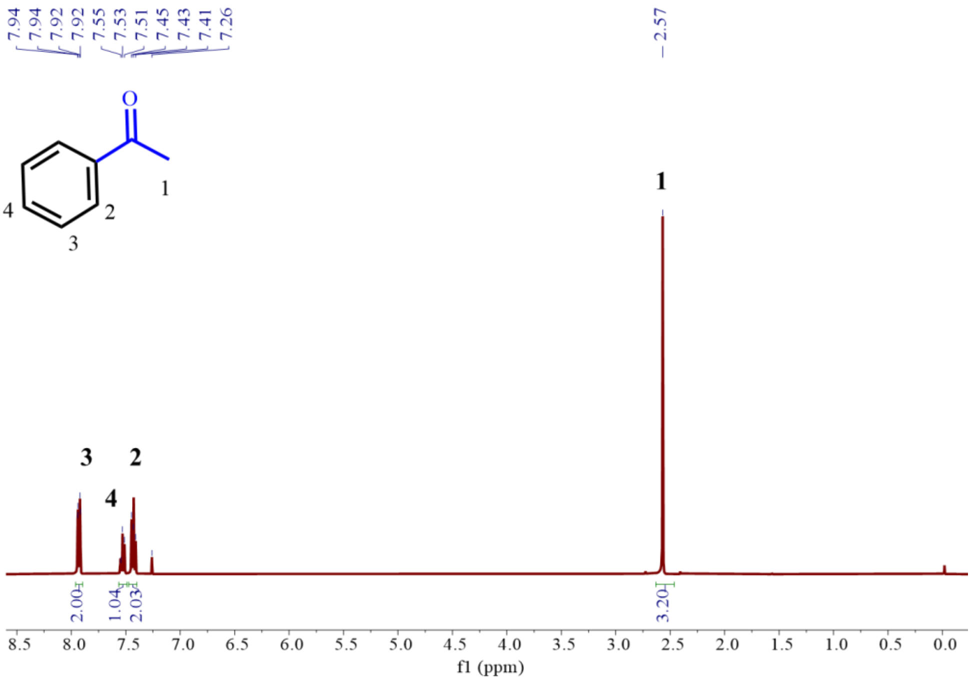

Figure S13. ^1H NMR spectrum (400 MHz, $CDCl_3$) δ 7.96 – 7.90 (m, 2H), 7.56 – 7.49 (m, 1H), 7.47 – 7.40 (m, 2H), 2.57 (s, 3H).

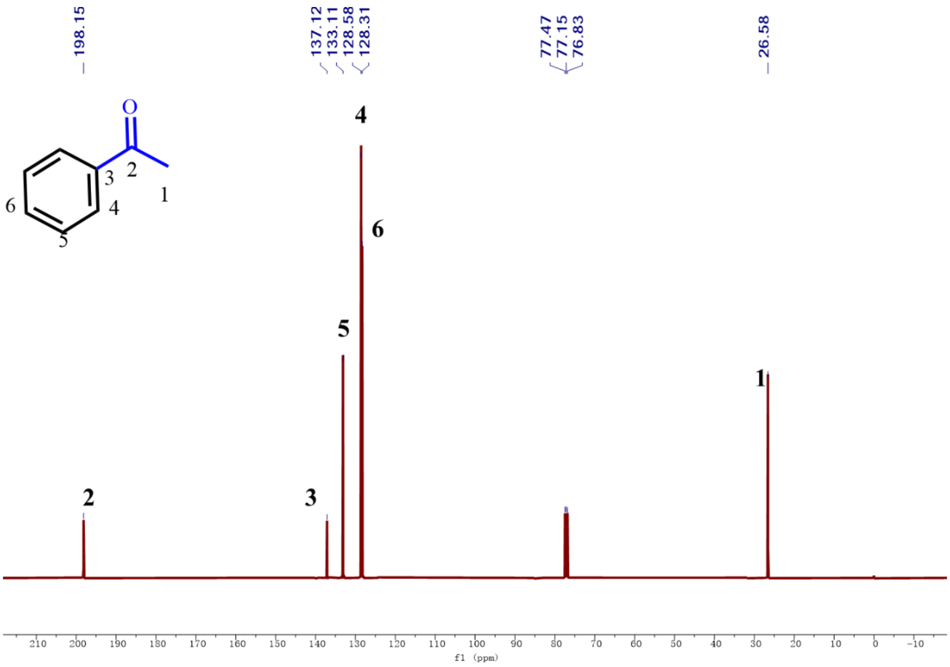

Figure S14. ^{13}C NMR spectrum (100 MHz, $CDCl_3$) δ 198.15, 137.12, 133.11, 128.58, 128.31, 77.47, 77.15, 76.83, 26.58.

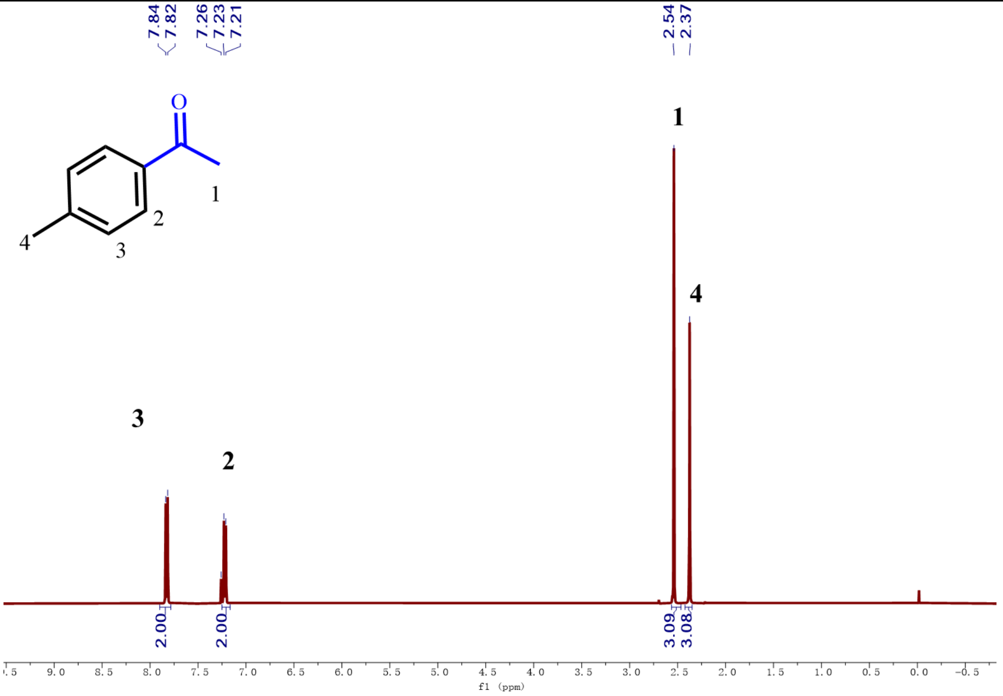

Figure S15. ^{1}H NMR spectrum (400 MHz, $CDCl_3$) δ 7.83 (d, *J* = 8.4 Hz, 2H), 7.22 (d, *J* = 7.8 Hz, 2H), 2.54 (s, 3H), 2.37 (s, 3H).

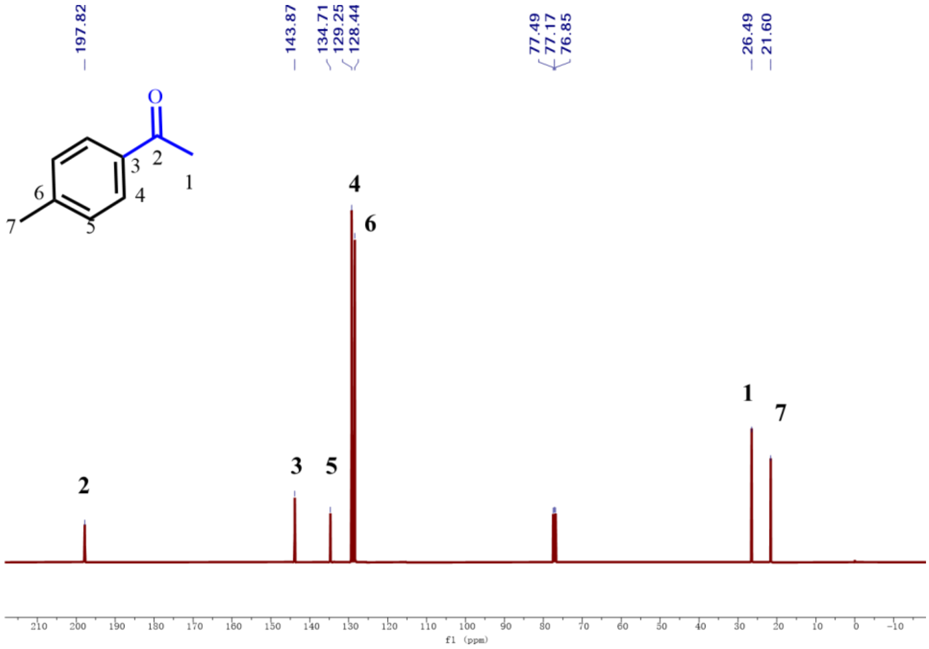

Figure S16. ^{13}C NMR spectrum (100 MHz, $CDCl_3$) δ 197.82, 143.87, 134.71, 129.25, 128.44, 77.49, 77.17, 76.85, 26.49, 21.60.

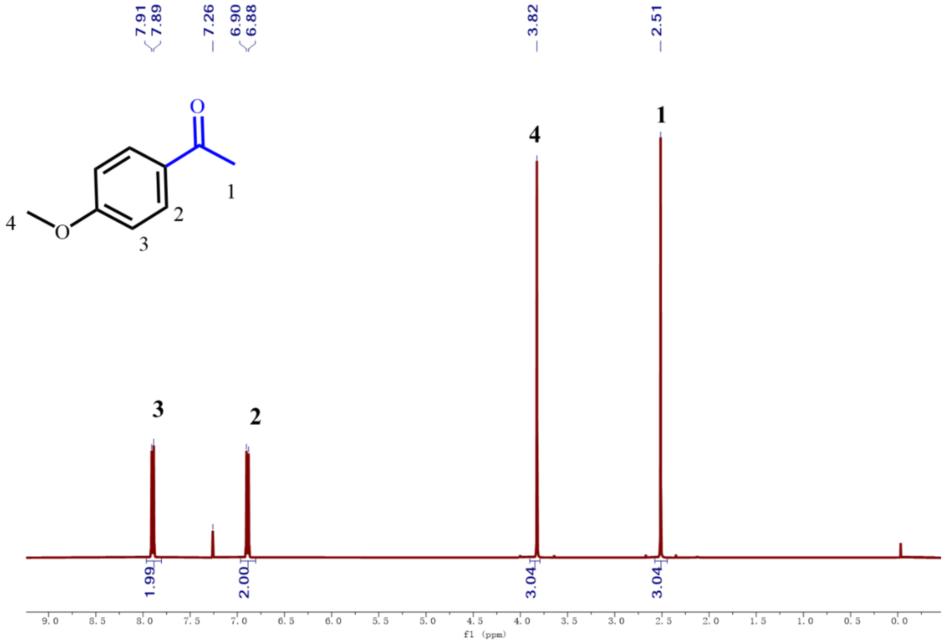

Figure S17. ^1H NMR spectrum (400 MHz, $CDCl_3$) δ 7.90 (d, *J* = 8.8 Hz, 2H), 6.89 (d, *J* = 8.8 Hz, 2H), 3.82 (s, 3H), 2.51 (s, 3H).

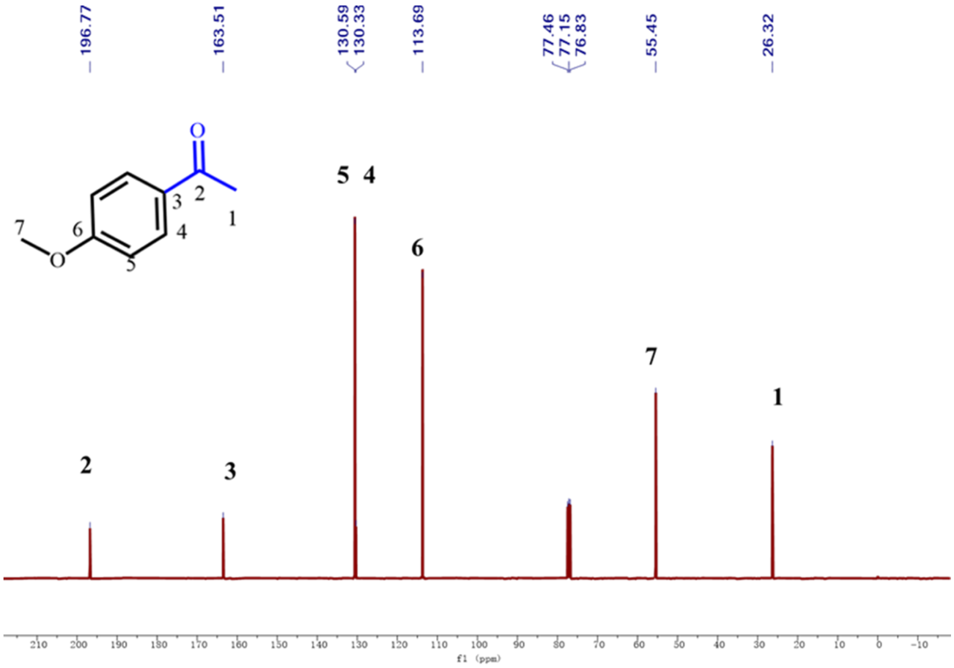

Figure S18. ^{13}C NMR spectrum (100 MHz, $CDCl_3$) δ 196.77, 163.51, 130.59, 130.33, 113.69, 77.46, 77.15, 76.83, 55.45, 26.32.

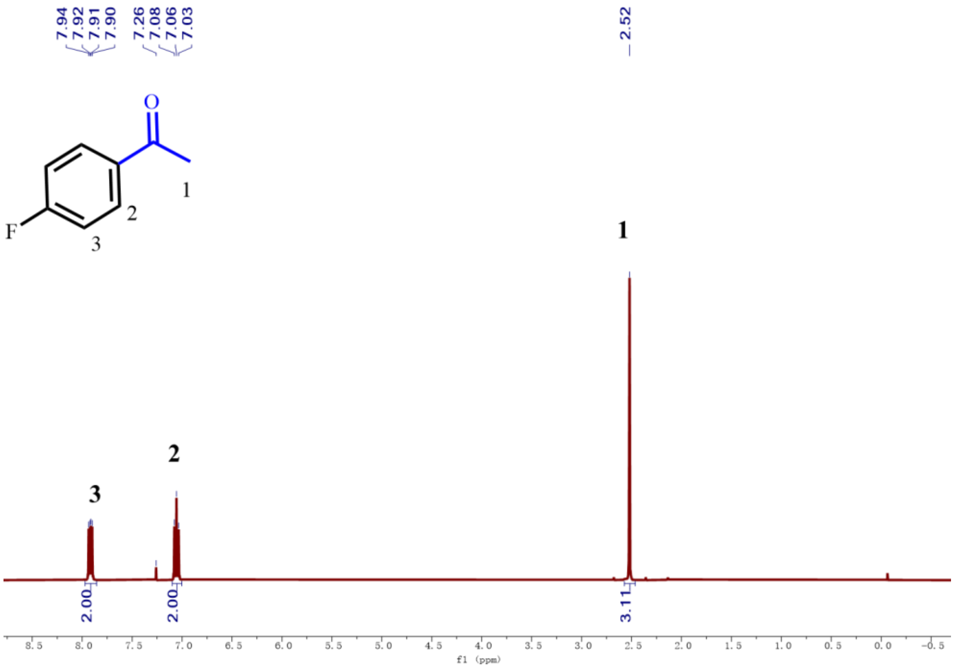

Figure S19. ^1H NMR spectrum (400 MHz, $CDCl_3$) δ 7.92 (dd, *J* = 8.8 Hz, 2H), 7.06 (t, *J* = 8.8 Hz, 2H), 2.52 (s, 3H).

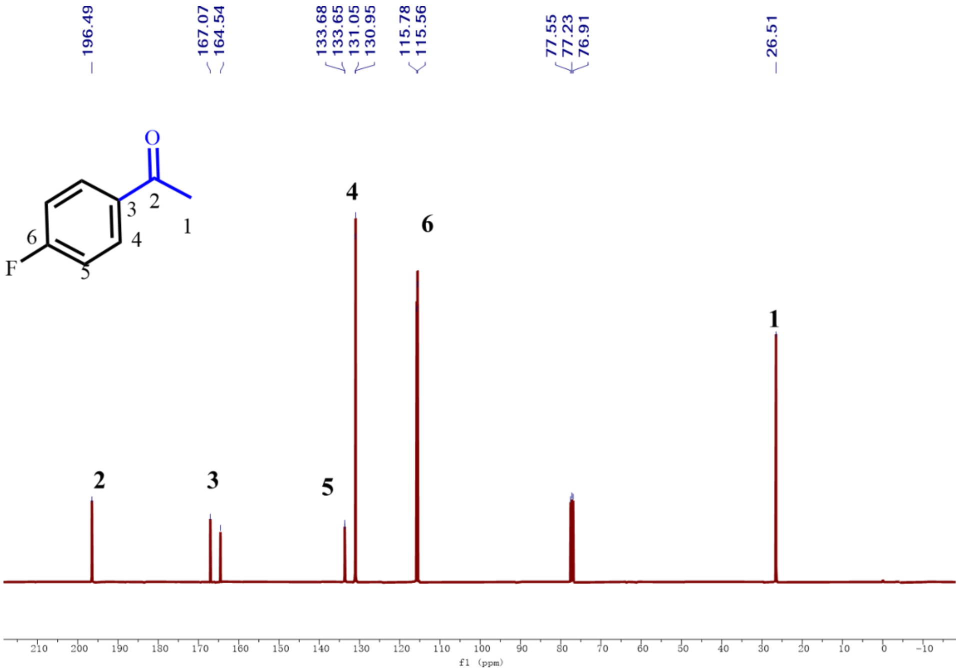

Figure S20. ^{13}C NMR spectrum (100 MHz, $CDCl_3$) δ 196.49, 167.07, 164.54, 133.68, 133.65, 131.05, 130.95, 115.78, 115.56, 77.55, 77.23, 76.91, 26.51.

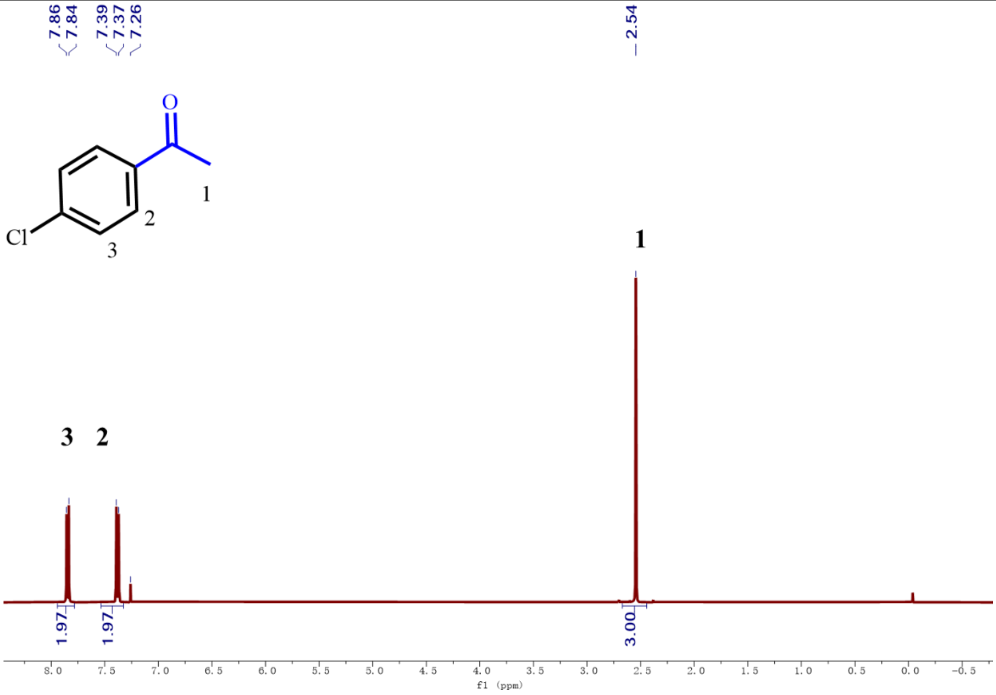

Figure S21. ^{1}H NMR spectrum (400 MHz, $CDCl_3$) δ 7.85 (d, *J* = 8.4 Hz, 2H), 7.38 (d, *J* = 8.4 Hz, 2H), 2.54 (s, 3H).

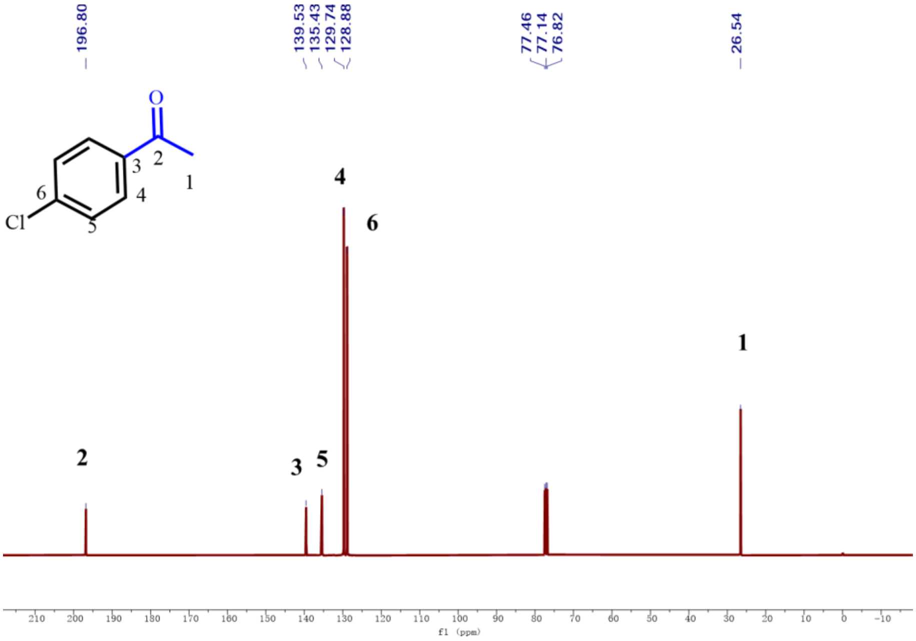

Figure S22. ^{13}C NMR spectrum (100 MHz, $CDCl_3$) δ 196.80, 139.53, 135.43, 129.74, 128.88, 77.46, 77.14, 76.82, 26.54.

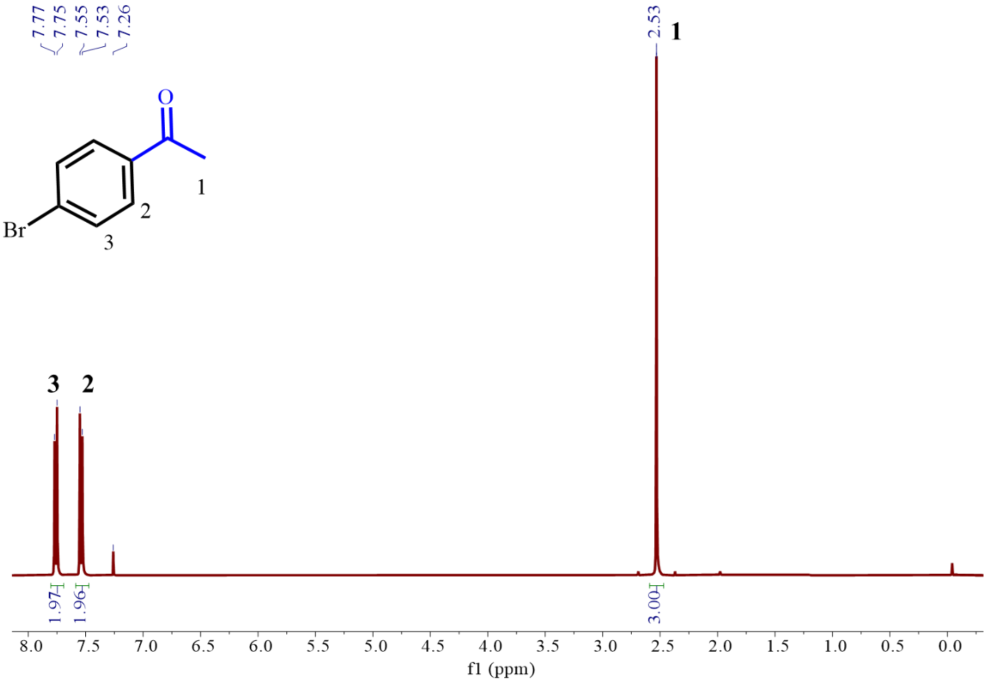

Figure S23. ^{1}H NMR spectrum (400 MHz, $CDCl_3$) δ 7.76 (d, *J* = 8.6 Hz, 2H), 7.54 (d, *J* = 8.6 Hz, 2H), 2.53 (s, 3H).

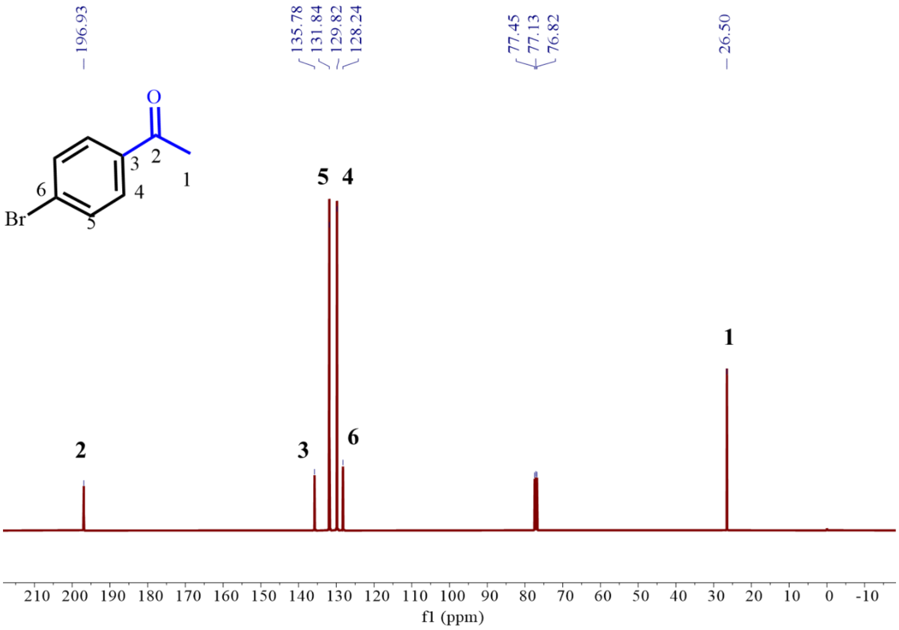

Figure S24. ^{13}C NMR spectrum (100 MHz, $CDCl_3$) δ 196.93, 135.78, 131.84, 129.82, 128.24, 77.45, 77.13, 76.82, 26.50.

polymers

MDPI

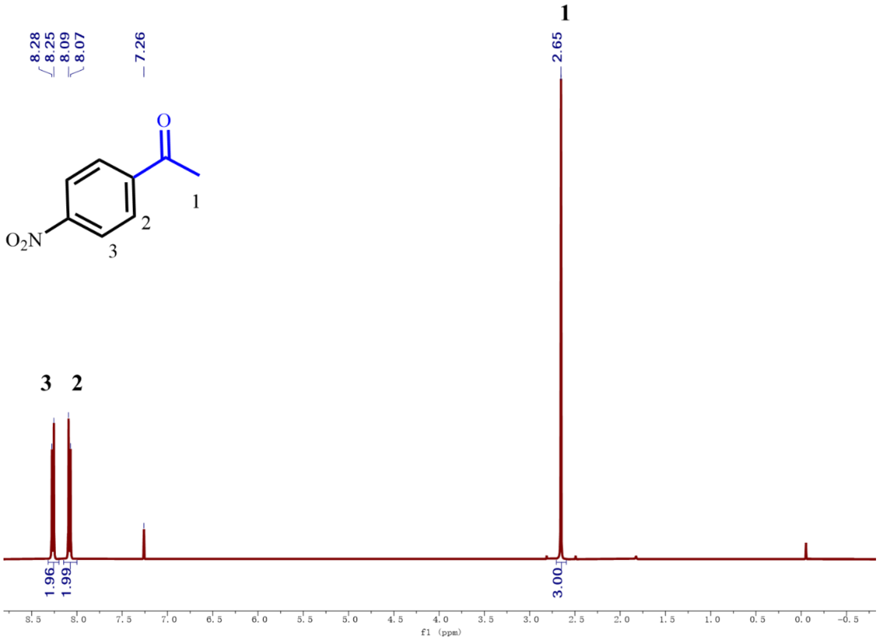

Figure S25. ^{1}H NMR spectrum (400 MHz, $CDCl_3$) δ 8.27 (d, *J* = 8.8 Hz, 2H), 8.08 (d, *J* = 8.8 Hz, 2H), 2.65 (s, 3H).

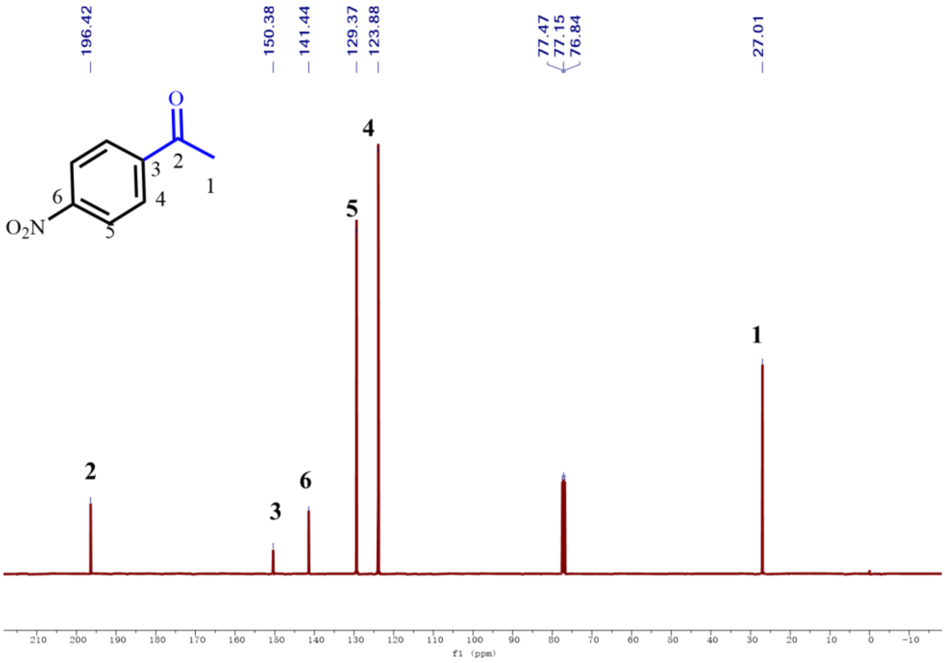

Figure S26. ^{13}C NMR spectrum (100 MHz, $CDCl_3$) δ 196.42, 150.38, 141.44, 129.37, 123.88, 77.47, 77.15, 76.84, 27.01.

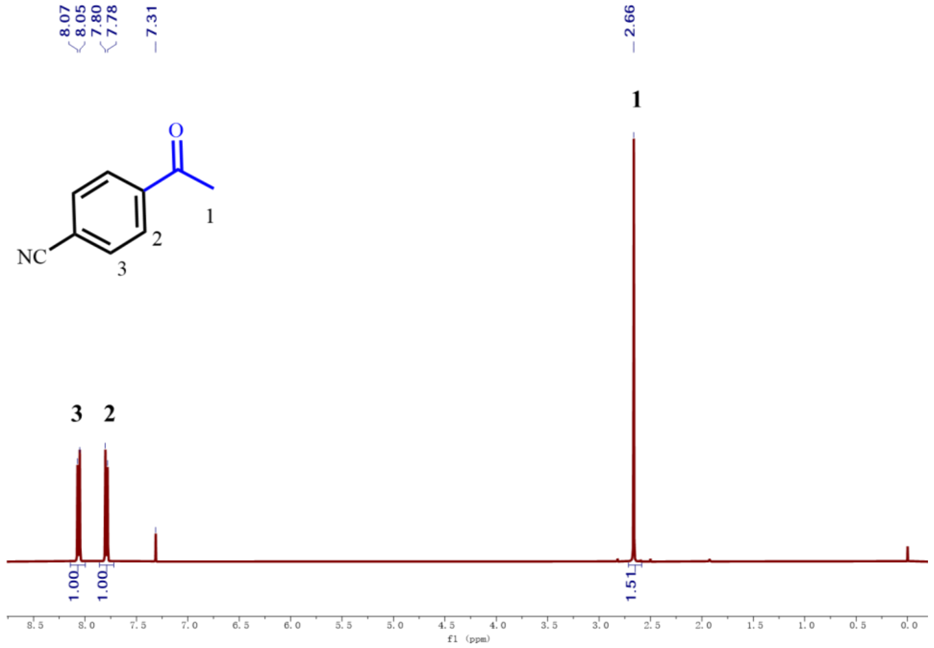

Figure S27. ^{1}H NMR spectrum (400 MHz, $CDCl_3$) δ 8.06 (d, *J* = 8.4 Hz, 1H), 7.79 (d, *J* = 8.6 Hz, 1H), 2.66 (s, 2H).

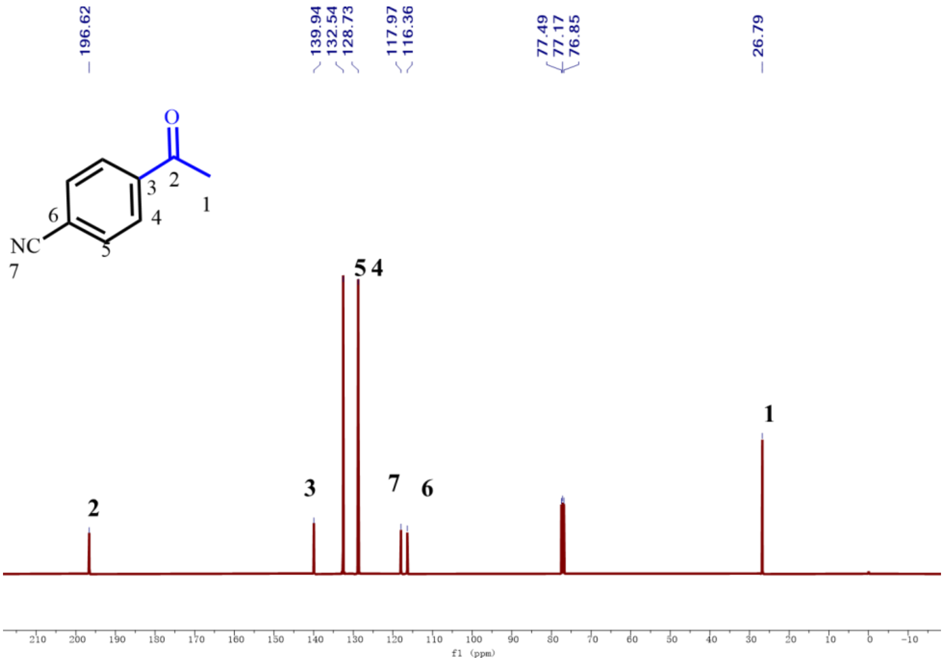

Figure S28. ^{13}C NMR spectrum (100 MHz, $CDCl_3$) δ 196.62, 139.94, 132.54, 128.73, 117.97 pm, 116.36, 77.49, 77.17, 76.85, 26.79
